# A geospatial analysis of flood risk zones in Cyprus: insights from statistical and multi-criteria decision analysis methods

**DOI:** 10.1007/s11356-024-33391-x

**Published:** 2024-04-26

**Authors:** Ma’in Abed Alhakim Naser Ghanem, Hasan Zaifoglu

**Affiliations:** 1 Sustainable Environment and Energy Systems, Middle East Technical University Northern Cyprus Campus, Orta Dogu Teknik Universitesi - Kuzey Kibris Kampusu, Guzelyurt Via Mersin 10, 99738 Kalkanli, Türkiye; 2 Civil Engineering Program, Middle East Technical University Northern Cyprus Campus, Orta Dogu Teknik Universitesi - Kuzey Kibris Kampusu, Guzelyurt Via Mersin 10, 99738 Kalkanli, Türkiye

**Keywords:** Flood risk map, Hazard, Vulnerability, Fuzzy AHP, Frequency ratio, Shannon’s entropy

## Abstract

Over the past few decades, flood disasters have emerged as the predominant natural hazard in Cyprus, primarily driven by the escalating influence of climate change in the Mediterranean region. In view of this, the objective of this study is to develop a geospatial flood risk map for the island of Cyprus by considering 14 flood hazard factors and five flood vulnerability factors, utilizing geographic information systems (GIS) and remotely sensed datasets. A comparative assessment was conducted for hazard mapping, employing statistical methods of frequency ratio (FR) and FR Shannon’s entropy (FR-SE), and multi-criteria decision analysis method of fuzzy analytic hierarchy process (F-AHP). The main findings indicated that the FR method exhibited the highest predictive capability, establishing it as the most suitable approach for flood hazard mapping. Additionally, vulnerability factors were aggregated using F-AHP to generate the vulnerability map. The resulting flood risk map, which is the product of flood hazard and flood vulnerability, revealed that 9% of the island was located within highly risky regions, while 13.2% was classified as moderate risk zones. Spatial analysis of these high-risk areas indicated their concentration in the primary city districts of the island. Therefore, to mitigate future risks within these cities, an analysis of potential expansion zones was conducted, identifying the best-suited zone exhibiting the lowest risk. The generated flood risk map can serve as a valuable resource for decision-makers on the island, facilitating the integration of flood risk analysis into urban management plans.

**Supplementary Information:**

The online version contains supplementary material available at 10.1007/s11356-024-33391-x.

## Introduction

Floods are considered to be among the most paramount natural disasters due to their widespread and devastating impacts. They can be categorized into different types, with flash floods and river floods being the most notable. Flash floods occur as a result of excessive rainfall events, leading to high discharge in a short period, while river floods cause an increase in water levels, leading to the inundation of surrounding areas. Globally, floods are the most frequently occurring natural disaster, accounting for 44% of all recorded natural disasters in the last 20 years, affecting approximately 1.65 billion people, resulting in over 100,000 deaths, and causing $651 billion in economic damage (Centre for Research on the Epidemiology of Disasters CRED and UN Office for Disaster Risk Reduction UNDRR 2020).

In Cyprus, over 330 flooding events were observed from 1971 to 2010. Of these, 5% are classified as high and very high in terms of their impacts on human health, the environment, cultural heritage, and economic damages, while 22%, 31%, and 42% were classified as mediocre, low, and very low, respectively (Kythreotou and Mesimeris 2022). Severe flood events have recently been witnessed over the island, including the 2010 and 2014 floods in the northern part of Nicosia, which caused the inundation of intercity roads, hospitals, and schools (Zaifoğlu 2018). The 2018 flood in Kyrenia also resulted in four fatalities. Moreover, studies on climate change indicate that extreme precipitation events are projected to intensify and become more frequent on daily and sub-daily scales (Zhang et al. 2021). Climate projections in the eastern Mediterranean and Cyprus consistently conclude that there will be a consistent increase in average, maximum, and minimum temperatures up to 2100; a decrease in annual precipitation leading to a drier climate; as well as variable changes in extreme precipitation indices (Hadjinicolaou et al. 2011; Mathbout et al. 2018). Furthermore, changes in population and land cover are increasing the vulnerability of areas to flooding and boosting the risks of flooding (Zope et al. 2016). Therefore, it is imperative to implement a sustainable flood risk management system that can adapt to these continuous changes and provide remedial measures.

Flood risk management is a critical process that provides practical solutions and increases public awareness of floods (Lin et al. 2020). For existing systems, flood risk management comprises four steps: risk analysis, maintenance development, preparedness, and disaster response (Plate 2002). Risk analysis involves combining hazard with vulnerability analysis to determine the general risk in a study area (Ramkar and Yadav 2021), which assists in making long-term planning decisions. Risk analysis can be conducted using four different methods: historical disaster statistical methods (Van Steenbergen et al. 2012), index system methods (Zhang et al. 2020), scenario simulation analysis (Alfieri et al. 2015), and geographic information systems (GIS)-based risk assessment (Lyu et al. 2018). GIS-based methods are becoming increasingly popular because of their versatility in data processing, spatial modeling, data analysis, visualization, and management. The availability of remotely sensed data enhances adaptability to data scarcity conditions, which can also support the creation of hydrological models in a GIS environment with ease (Wang and Xie 2018). Flood risk mapping in GIS is often performed on a basin scale (Hasanuzzaman et al. 2023), particularly in morphometric risk studies (Khalifa et al. 2022), which use various stream and basin factors to provide a comparative flood risk map among basins, and on a grid scale, where a risk score is calculated for each grid in the area of interest (Lyu and Yin 2023).

Various methodologies have been implemented in grid scale mapping of flood hazard, vulnerability, and risk in a GIS environment. The multi-criteria decision analysis (MCDA) method combined with the analytical hierarchy process (AHP) has been widely employed to calculate the relative weights of the parameters and generate the flood hazard map (Kittipongvises et al. 2020). For example, Ganji et al. (2022) used the fuzzy AHP (F-AHP) method to create the flood hazard map. Sarkar et al. (2022) utilized two common techniques, namely, the frequency ratio (FR) and FR Shannon’s entropy (FR-SE) methods, to assess flood hazard. Herein, the FR method uses historical flood point data and correlates them with the chosen flood inducing parameters. The SEI method, also known as a modified frequency ratio method, calculates the relationship between the flood-inducing factors and the results through the entropy index. Other methods, including the ISO-maximum likelihood clustering algorithm (Lin et al. 2020), a hybrid F-AHP with decision-making trial and evaluation laboratory (Kanani-Sadat et al. 2019), and machine and deep learning techniques (Motta et al. 2021; Rafiei-Sardooi et al. 2021), have also been employed in the literature.

Flood risk assessment involves the analysis of two major types of factors: hazard and vulnerability. Hazard factors encompass natural and human-induced elements that contribute to flood occurrences (Motta et al. 2021). However, selecting hazard factors lack universally accepted criteria in literature. Therefore, flood hazard studies rely upon the literature to identify the flood-predicting factors in terms of their number and influence. These factors should consider the meteorological, hydrological, geomorphological, and topographic conditions in the study area. They should also be independent of each other and successfully predict flood occurrence (Mudashiru et al. 2021). Additionally, depending on the regional circumstances and data accessibility, these factors may change. Slope, distance from streams, drainage density, elevation, soil map, curve number, precipitation, flow accumulation, topographic wetness index, and stream power index are some of the factors that are frequently utilized for flood hazard mapping (Dash and Sar 2020; Allafta and Opp 2021; Chukwuma et al. 2021). On the other hand, flood vulnerability refers to conditions influenced by various physical, social, economic, and environmental factors that increase the vulnerability to hazards (Rincón et al. 2018; Darabi et al. 2019). Age, education, income, population density, road network, land use, and land cover are just a few of the factors that are considered when mapping flood vulnerability (Xiong et al. 2019; Hussain et al. 2021; Ekmekcioğlu et al. 2021). By combining the maps of flood hazard and vulnerability, the flood risk map in this study is created, considering both the likelihood that a hazard would occur and its possible effects (Pham et al. 2021).

The main objective of this study is to identify suitable factors related to flood hazard and vulnerability in order to generate a comprehensive flood risk map for the island of Cyprus. To achieve this, a range of hazard and vulnerability factors were collected utilizing geographic information systems and remote sensing datasets. These factors were integrated using statistical methods of FR and FR-SE, and the multi-criteria decision analysis method of F-AHP to create hazard and vulnerability maps. Furthermore, the predictive capabilities of the aforementioned methods were tested with the receiver operating characteristic (ROC) curves. Finally, the flood risk map was developed by combining the highest predictability flood hazard map with the flood vulnerability map, emphasizing its significance in assisting stakeholders and decision-makers protecting the population, mitigating economic losses, and promoting sustainable water management practices.

## Materials and methods

### Study area

**Fig. 1:**
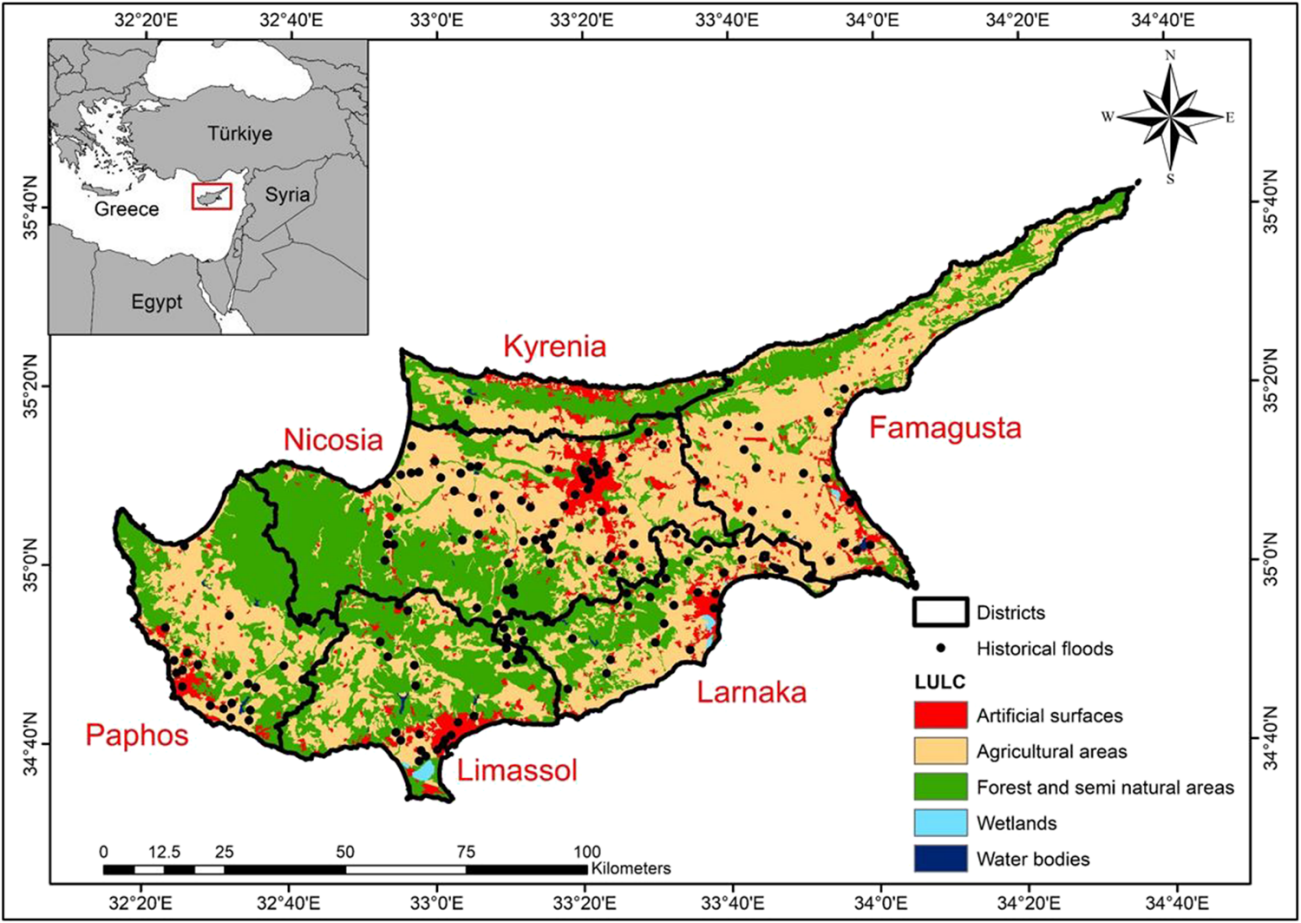
Location of the island of Cyprus

The island of Cyprus, positioned between the longitudinal coordinates of 32°24′43.59″–34°35′20.39″ and latitudinal coordinates of 34°45′15″–35°41′49″, is the third largest island in the Mediterranean Sea with an area of 9251 km^2^. The island experiences typical Mediterranean weather, with hot, dry summers and warm, rainy winters. The average annual rainfall of 500 mm on the island of Cyprus exhibits variations influenced by its diverse topography, including the Troodos and Kyrenia Mountains, the Mesaoria plain located in the central region, and the Karpass Peninsula in the northeast. While the Troodos mountains receive up to 1000 mm of precipitation annually, the central regions of the island have an average of approximately 300 mm. Rainfall is concentrated between October and March, with December, January, and February being the wettest months (Hadjinicolaou et al. 2011; Cyprus Meteorological Survey CIY 2023). The streams on the island originate from the Troodos and Kyrenia mountain ranges. The seasonal variation in rainfall leads to the ephemeral nature of streams on the island. In addition, the island of Cyprus comprises 48% agricultural lands, 42% forests and semi-natural forests, 1% water bodies and marshes, and 9% artificial surfaces, distributed across the six major districts of Nicosia, Paphos, Larnaca, Limassol, Kyrenia, and Famagusta, with a total population of approximately 1.25 million people as of 2020 (United Nations 2022). The Water Development Department of the Republic of Cyprus identified 154 historical flood locations, which have been documented and digitized on the island based on Directive 2007/60/EC of the European Union (The General Civil Defense Administration Cyprus GEDPA 2022). A map displaying the island of Cyprus, its districts, and historical flood locations is presented in Fig. 1. Additionally, it illustrates the remotely sensed CORINE Land Use/Land cover (LULC) for 2018 (Büttner et al. 2021).

### Datasets

The flood risk map generated in this study was developed using datasets that integrated hazard (H) and vulnerability (V) factors. The literature commonly classifies hazard parameters into three distinct categories: meteorological factors (such as precipitation and extreme rainfall indices), topographic factors resulting from variations in elevation (such as slope), and hydrologic parameters denoting the hydrologic response (Ma et al. 2021). In addition, the morphometric risk is considered a general parameter within hazard factors, as it encompasses scale, basin shape, topographic, and hydrologic parameters. The vulnerability factors are classified as exposure to hazard factors and coping capacity factors that depend on the capability of local defense systems (Chen and Alexander 2022). The selected factors for hazard and vulnerability mapping are presented in Table 1, where 14 factors were chosen for flood hazard mapping and five factors were chosen for vulnerability mapping.

**Table 1:** Data sources and factors used in hazard and vulnerability maps

Factor category	Factor class	Factor	Symbol	Resolution	Source	Temporal coverage
Meteorological (M)	Hazard	Mean annual rainfall	AR	5 km	CHIRPS	1981–2022
		Very heavy precipitation days (precipitation > = 20 mm)	R20	5 km	CHIRPS	1981–2022
Topographic (T)	Hazard	Digital elevation model	DEM	30 m	AW3D30	––-
		Slope	SL	30 m	GIS generated	––-
		Aspect	AS	30 m	GIS generated	––-
		Profile curvature	ProfC	30 m	GIS generated	––-
		Plan curvature	PlanC	30 m	GIS generated	––-
Hydrologic (H)	Hazard	Topographic wetness index	TWI	30 m	GIS generated	––-
		Stream power index	SPI	30 m	GIS generated	––-
		Distance from river	DFR	30 m	GIS generated	––-
		Curve number	CN	250 m	CORINE	2018
					HYSOGs250m	
					TR-55	
		Overland flow time	TC	100 m	GIS generated	2018
		Relative reservoir area	RRA	––-	CWP	2022
General (G)	Hazard	Morphometric risk	MR	––-	GIS generated	––-
Exposure (E)	Vulnerability	Population	P	100 m	WPG	2020
		Percentage change in artificial surfaces	UR	––-	CORINE	2012–2018
		Economic value of buildings	EV	10 m	Cystat	2020
Coping capacity (C)	Vulnerability	Road vulnerability index	RVI	30 m	OSM	2022
		Distance from hospitals	DFH	30 m	OSM	2022

#### Hazard factors

**Fig. 2:**
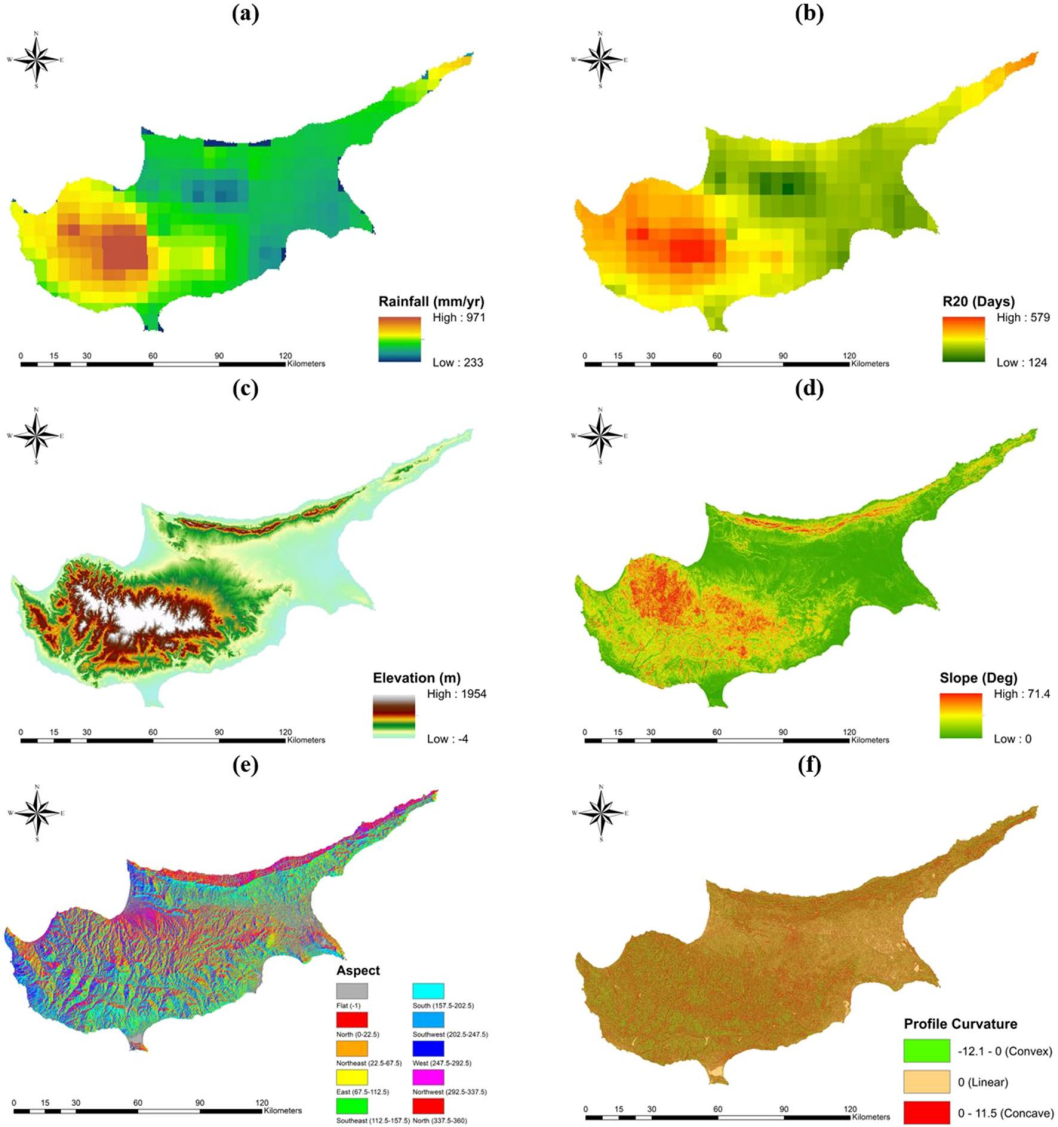

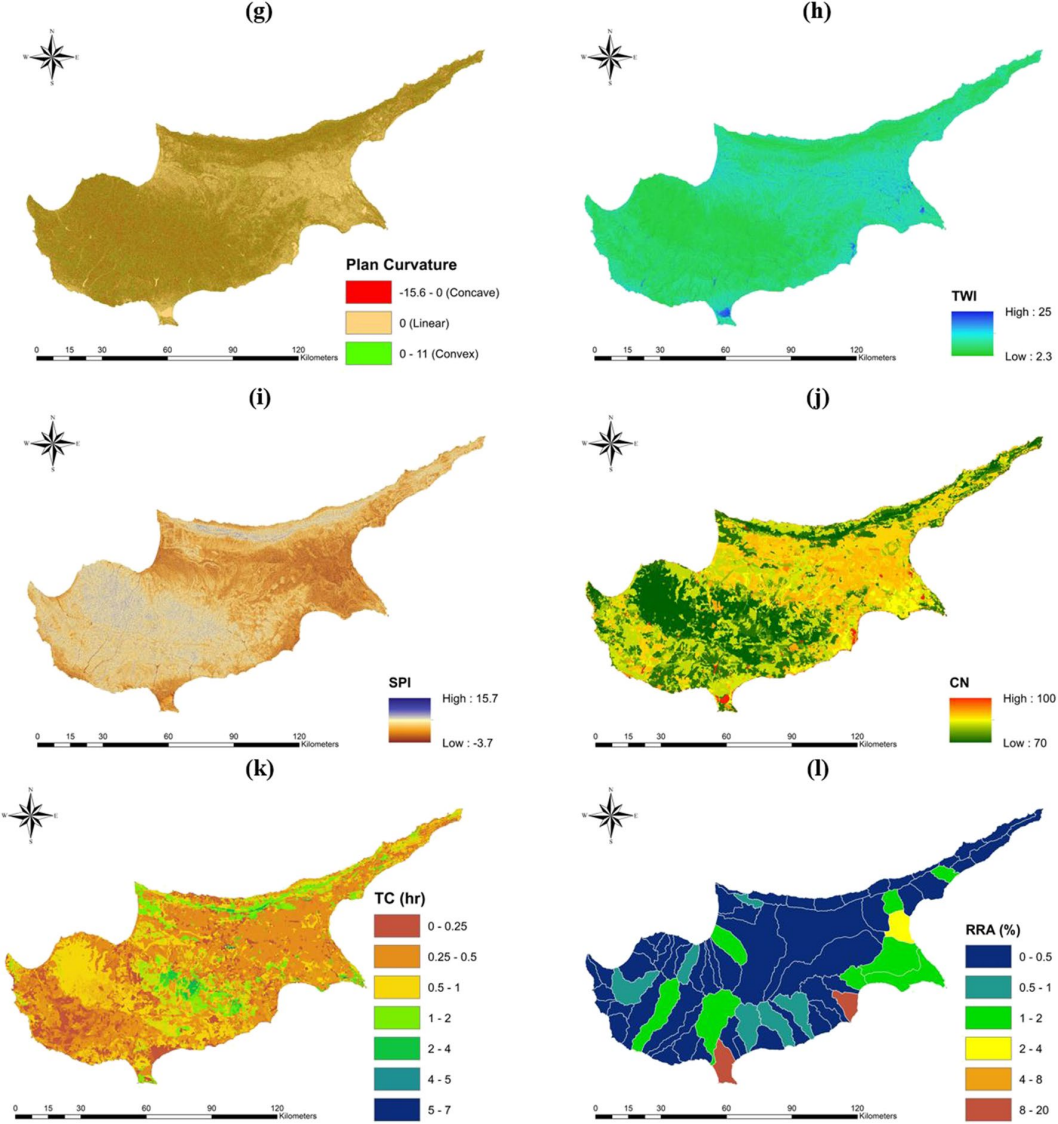
Flood hazard factors: **a** AR, **b** R20, **c** DEM, **d** SL, **e** AS, **f** ProfC, **g** PlanC, **h** TWI, **i** SPI, **j** CN, **k** TC, and **l** RRA

The meteorological factors were obtained using the CHIRPS (Climate Hazards Group InfraRed Precipitation with Station data) dataset (Funk et al. 2014). This dataset was selected due to its high correlation coefficients with station measurements and its spatial coverage of the entire island (Funk et al. 2014; Katsanos et al. 2016a). Meteorological factors, specifically the annual rainfall and extreme precipitation indices, have a direct impact on flooding. Higher annual rainfall indicates greater flood hazard potential in the long run (Fig. 2a), while the extreme precipitation indices of R20 (Fig. 2b) and R99p provide valuable information on the probability and severity of flooding events (Katsanos et al. 2016b).

The topographic factors utilized in the analysis were derived from the ALOS World 3D-30m (AW3D30) dataset (Tadono et al. 2014). Elevation (Fig. 2c) was found to be a critical parameter in flood potential mapping, as lower elevation areas were found to be more prone to flooding (Parsian et al. 2021). In contrast, slope (Fig. 2d) was observed to have an inverse relationship with flood potential, with flatter regions posing a higher risk of water accumulation (Radwan et al. 2019). The aspect (Fig. 2e) map displays the direction and degree of steepness of the slope and is commonly used as a flood conditioning factor, which was classified into the common aspect directions found in the literature (Bhatt et al. 2014). Profile curvature (Fig. 2f) was found to impact the acceleration of water on a surface and was classified into three classes: upwardly convex, linear, and upwardly concave, with the latter causing the highest acceleration in water flow (Khosravi et al. 2020). Plan curvature (Fig. 2g) indicates the sideward direction of the surface, affecting the convergence of the flow. Negative values indicate that the surface is laterally concave, thereby increasing flood risk by converging the flow (Malik et al. 2020). Additionally, a lower distance from the river was observed to increase the risk of inundation due to river floods (Pathan et al. 2022).

An indicator of soil moisture and flow accumulation capacity, the topographic wetness index, TWI (Fig. 2h), was obtained to understand the effect of topography on hydrologic processes. Higher values indicate higher accumulation and flood potential (Saha and Agrawal 2020). Besides, the stream power index, SPI (Fig. 2i), functioned as a measure of the erosive force of water flow, with higher SPI values indicating higher flow velocities (Mojaddadi et al. 2017). The following formulas are used to determine TWI and SPI:

1 $${\text{TWI}}={\text{ln}}\left( \frac{\alpha }{{\text{tan}}\beta }\right)$$

2 $${\text{SPI}}={\text{ln}}(\alpha {\text{tan}}\beta )$$

where $\alpha$ is the upstream catchment area per unit contour length and $\beta$ is the local slope in radians. Furthermore, the soil conservation service-curve number (SCS-CN) method was employed to compute and measure the rainfall-runoff response of a specific basin. This method combines data on land use, land cover, soil type, and soil texture to determine the CN value (Singh et al. 2021). In this study, the remotely sensed datasets of CORINE LULC 2018 level 3 map (Büttner et al. 2021) and the Global Hydrologic Soil Group (HYSOGs250m) map (Ross et al. 2018) were combined using GIS techniques. Following that, the CN values were assigned, considering 37 different LULC types, 4 soil texture types, and the standard CN values from Urban Hydrology for Small Watersheds TR-55 (TR-55), assuming average antecedent wet conditions (Cronshey 1986). The detailed Corine LULC map, soil texture map, and average CN values for different LULC types are presented in S.M 1. The obtained CN values for the study area ranged between 70 and 100, with the latter assigned to water surfaces (Fig. 2j). Moreover, the flow time was measured by estimating the overland time of concentration within each grid cell. This was achieved using the steady-state kinematic wave approximation, with the Manning equation being utilized to account for the roughness of the flow path (Melesse and Graham 2004):

3 $$TC=\frac{{L}^{0.6} {n}^{0.6}}{{i}_{e}^{0.4}{ SL}^{0.3}}$$

where $L$ is length of the flow inside the grid cell (m), $n$ is the Manning’s roughness coefficient, ${i}_{e}$ is the average excess rainfall intensity (m/s), and $SL$ is the slope of the grid cell in percent rise. The length of overland flow varies depending on the flow direction, either diagonal or horizontal/vertical. The Manning’s roughness coefficients are assigned to CORINE LULC classes, using recommended Manning’s roughness tables in the literature (Papaioannou et al. 2018; Lorenzo-Lacruz et al. 2019), and can be inspected in S.M 1. Moreover, average excess rainfall intensity is measured as the total depth of excess rainfall per rainfall duration (Ponce and Hawkins 1996):

4 $${i}_{e}=\frac{{P}_{e}}{T}$$

where

5 $${P}_{e}=\frac{{\left(P-0.2{S}_{p}\right)}^{2}}{\left(P+0.8{S}_{p}\right)}$$

where

6 $${S}_{p}=\frac{25400}{{\text{CN}}}-254$$

Herein, $T$ is the duration of rainfall (s), $P$ is the total depth of rainfall (mm), and ${S}_{p}$ is the potential maximum retention (mm). In the study, the value for $P$ was determined as the 99th percentile of rainfall. The parameter was computed from the CHIRPS satellite rainfall data, with a spatial resolution of 5 km and temporal coverage from 1981 to 2022. The selection of CHIRPS for computing the 99th percentile was based on its strong correlation coefficient of 0.75, as identified in a previous study conducted in Cyprus (Katsanos et al. 2016b). The duration of rainfall aggregation in CHIRPS data was selected as 1 day and denoted as $T$. The flow time (Fig. 2k) on the island exhibited substantial variation, ranging from almost 0 to 7 h, depending on the local topography, rainfall, CN, and roughness coefficients. Furthermore, the relative reservoir ratio (RRA) represents the percentage of water-retaining structures within a hydrologic basin (Adnan et al. 2019). Reservoir capacities were obtained from the Cyprus Wetlands Project (CWP) conducted by Terra Cypria, the Cyprus Conservation Foundation (Terra Cypria 2022). A higher RRA percentage indicates the presence of more water control structures in the basin, which reduces the hazard potential. The obtained values of RRA (Fig. 2l) ranged from nearly 0 to 20% in basins containing large lakes and other water-retaining structures.

#### Vulnerability factors

**Fig. 3:**
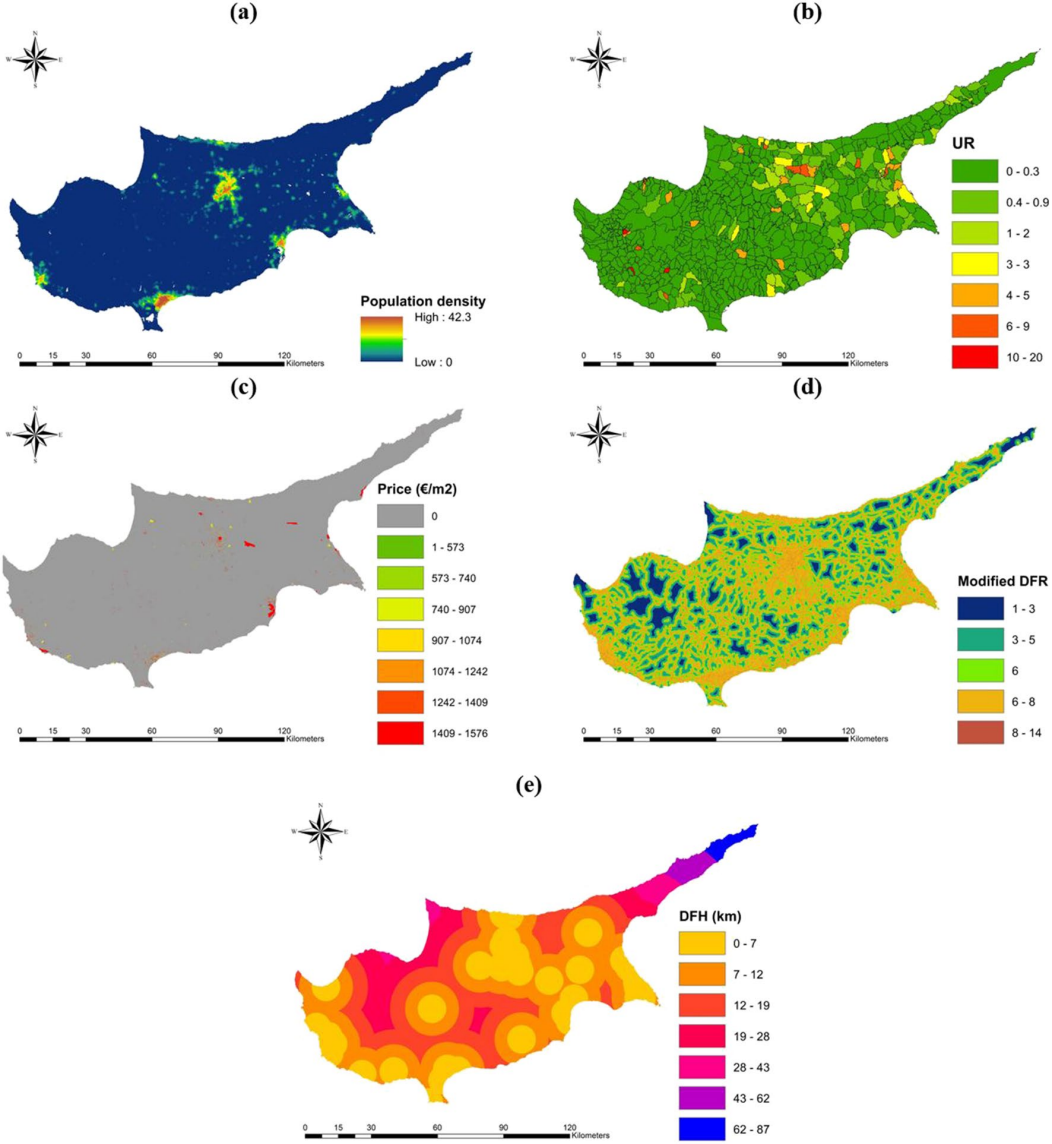
Flood vulnerability factors: **a** P, **b** ${\text{UR}}$, **c** ${\text{EV}}$, **d** ${\text{RVI}}$, and **e** ${\text{DFH}}$

Population density, as illustrated in Fig. 3a, is a crucial social exposure factor in the context of hazard risk assessment (Dandapat and Panda 2017). Areas with higher population densities are associated with a greater risk of exposure (Pathan et al. 2022). The population data used in this study were derived from a comparative analysis of remotely sensed population density sources, coupled with the initial results of the first administrative level population census conducted in 2021. Accuracy indices, including relative error, weighted area error, and total population error, were utilized to evaluate the precision of the population datasets (Yang et al. 2013). For the year 2020, the WorldPop Global (WPG) unconstrained datasets (Lloyd et al. 2019) were selected due to their relative error of 1.69%, weighted area error of 1.7%, and total population error of 0.09%. Particularly, population density is negligible in remote areas, but in major cities, it can reach up to 43 people per 100 m^2^, thus increasing the risk of exposure to hazards.

To account for urbanization, the percentage increase in artificial surfaces was computed for third-level administrative villages, as shown in Fig. 3b (Islam et al. 2021). The urbanization rate index (${\text{UR}}$) was utilized to gauge the relative growth of artificial man-made surfaces within a given urban area. Since man-made surfaces have higher runoff coefficients than natural surfaces, villages with higher ${\text{UR}}$ values are considered to be at a greater risk of hazard exposure. The calculation of ${\text{UR}}$ is based on the following formula:

7 $${\text{UR}}=\frac{A{S}_{2012-2018}*100}{{A}_{v}}$$

where $A{S}_{2012-2018}$ is the area of artificial surfaces built in the 2012–2018 period and ${A}_{v}$ is the village area. ${\text{UR}}$ ranges from 0 in the villages without condensed urbanization to 16.81% in the riskier villages like Gerovasa, with most of the villages having ${\text{UR}}$ values within 0–2%. Floods are a major threat to buildings and can cause damage and potential structural failure, resulting in economic damages (Nadal et al. 2010).

The local prices of construction per m^2^ for various building types, as depicted in Fig. 3c, were obtained from the Statistical Service of Cyprus (Cystat) (Cyprus Statistical Service Cystat 2022) and linked with open street map building data. The economic value, EV, which denotes the average cost of construction, varies significantly across different building types. For instance, the building costs for industrial, service, tourist service, dwellings, and churches are 678, 1297, 1222, 1024, and 1576 €/m^2^, respectively. Moreover, the distance from roads is a crucial factor that indicates the vulnerability of the road network to floods, owing to road damage and mobility issues (Sukcharoen et al. 2016). In this study, the OpenStreetMap (OSM) served as a data source for roads (Bennet 2010; Geofabrik 2022). The distance from roads was computed using the Euclidean distance function in GIS and classified into seven categories using the natural breaks method. The road importance factor was assigned to roads based on the country-wise road importance scale from open street map. Residential, unclassified, tertiary, secondary, primary, trunk, and motorways were scaled from 1 to 7, respectively, according to their importance. Subsequently, the road vulnerability index (RVI) was computed by summing the distance from roads and the road importance factor. As shown in Fig. 3d, the RVI values range from 1 to 14, with 14 representing the highest vulnerability. Furthermore, the distance from hospitals (DFH) serves as a coping capacity factor that measures the ability to transfer potential patients during a flood event (Ahmadi et al. 2023). As seen in Fig. 3e, the index values range from 0 to a maximum of 87 km in the Karpas Peninsula, making it the most vulnerable region on the island.

### Methods

**Fig. 4:**
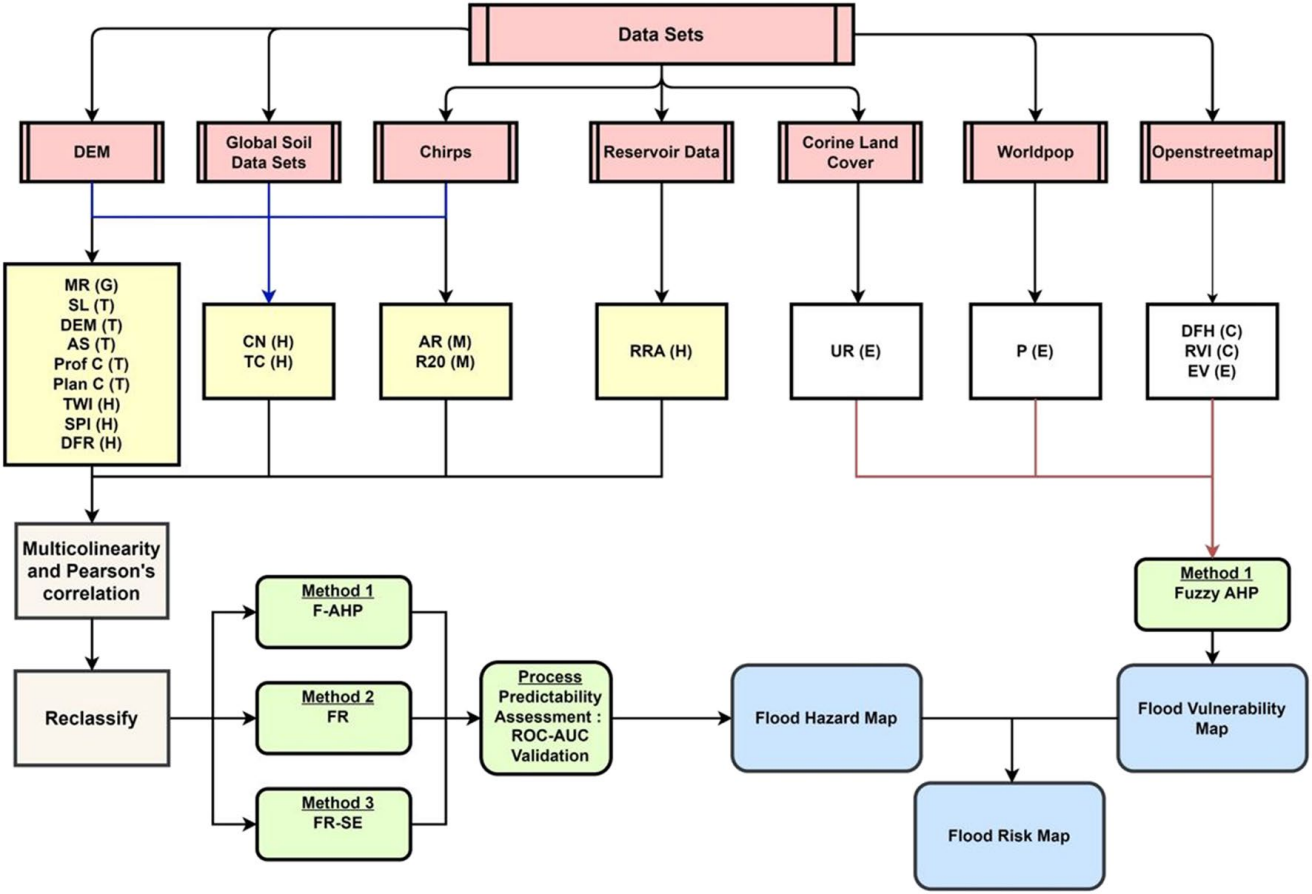
Flood risk mapping flow chart. Pink, data source; yellow, hazard factor (minor symbol); white, vulnerability factor (minor symbol); gray, data preparation step; green, testing method; blue, results

The schematic illustration of the applied methodology is presented in Fig. 4 and comprises the following major steps: (i) identification of remote sensing datasets, (ii) computation of flood hazard and flood vulnerability factors, (iii) utilization of statistical and/or MCDM techniques to generate flood hazard and flood vulnerability maps, and (iv) generation of a comprehensive flood risk map for the island of Cyprus.

#### Morphometric risk

Morphometric analysis is a widely used methodology in the literature and aims to understand the characteristics of basins and streams through the interpretation of calculated parameters and mapping of the total flood risk (Nasir et al. 2020). The basins in Cyprus were delineated according to the study conducted for Cyprus under the EU Water Directive Framework (Ministry of Agriculture, Natural Resources and Environment 2005). These delineated basins were then utilized for morphometric analysis in this study. In this context, firstly, the digital elevation model was filled in GIS environment to remove the sinks. Then, flow direction and flow accumulation maps were obtained and streams were delineated with a minimum flow accumulation threshold of 100 (Youssef et al. 2011), which was used to calculate the stream orders. The available streams and basins are then used to calculate the parameters listed in Table 2. All normalized parameters within the basin are added to generate the total risk associated with the basins. The resultant values are further normalized between the basins to create the morphometric risk map, with a scale ranging from 1 to 5.

**Table 2:** Morphometric analysis parameters

Parameter	Symbol	Equation/explanation	Correlation	Source
Scale parameters				
Basin area (km^2^)	*A*	Area of the watershed	Positive (Abdel-Fattah et al. 2017)	Horton (1932)
Perimeter (km)	*P*	Perimeter of the watershed	Positive (Abdel-Fattah et al. 2017)	Schumm (1956)
Basin length (km)	*L*_b_	Maximum basin length	Positive (Bajabaa et al. 2014)	Schumm (1956)
Width/length ratio	*WL*	*WL* = *W*/*L*_b_	Negative (Akay and Baduna Koçyiğit 2020)	Schumm (1956)
Topographic parameters				
Total basin relief (m)	*R*	*R* = (*H*_max_ − *H*_min_)	Positive (Aher et al. 2014)	Hadley and Schumm (1961)
Relief ratio	*R*_hl_	*R*_hl_ = *R*/ *L*_b_	Positive (Aher et al. 2014)	Schumm (1956)
Ruggedness number	*R*_n_	*R*_n_ = *D*_d_(*R*/1000)	Positive (Bajabaa et al. 2014)	Melton (1957)
Shape parameters				
Form factor	*F*	*F* = *A*/*L*_b_^2^	Positive (Alqahtani and Qaddah 2019)	Horton (1932)
Compactness ratio	*C*	*C* = *P*/2√(*πA*)	Negative (Youssef et al. 2011)	Horton (1932)
Circularity ratio	*R*_c_	*R*_c_ = 4*πA*/*P*^2^	Positive (Alqahtani and Qaddah 2019)	Schumm (1956)
Elongation ratio	*R*_e_	2√(*A*/*π*/*L*_b_)	Negative (Alqahtani and Qaddah 2019)	Schumm (1956)
Drainage network parameters				
Stream order	*U*	Number of stream orders in each watershed	Positive (Youssef et al. 2011)	Strahler (1952)
Stream number	*N*_u_	Total stream number of each watershed Total stream length of each watershed	Positive (Youssef et al. 2011)	Strahler (1952)
Total stream length (km)	*L*_u_		Positive (Bajabaa et al. 2014)	Horton (1945)
Stream frequency (1/km^2^)	*F*_s_	*F*_s_ = *N*_*u*_/*A*	Positive (Taha et al. 2017)	Horton (1945)
Drainage density (1/km)	Dd	*D*_d_ = *L*_*u*_/*A*	Positive (Taha et al. 2017)	Horton (1945)
Texture ratio (1/km)	*R*_t_	*R*_t_ = *N*_*u*_/*P*	Positive (Taha et al. 2017)	Horton (1945)
Bifurcation ratio	*R*_b_	*R*_b_ = *N*_*u*_/*N*_*u*+1_	Negative (Taha et al. 2017)	Horton (1945)

When a positive relationship exists between the computed parameter and morphometric risk, the normalization of the parameters (${Y}_{n}$) from 1 to 5 over the 70 basins is carried out using the following equation (Adnan et al. 2019):

8 $${Y}_{n}=\frac{\left({y}_{2}-{y}_{1}\right)\left({X}_{n}-{X}_{{\text{m}}\dot{{\text{i}}}{\text{n}}}\right)}{\left({X}_{{\text{max}}}-{X}_{{\text{m}}\dot{{\text{i}}}{\text{n}}}\right)}+{Y}_{1}$$

Besides, if there is a negative relationship, then normalization is performed as follows:

9 $${Y}_{n}=\frac{\left({y}_{2}-{y}_{1}\right)\left({X}_{n}-{X}_{max}\right)}{\left({X}_{{\text{m}}\dot{{\text{i}}}{\text{n}}}-{X}_{{\text{max}}}\right)}+{Y}_{1}$$

where ${y}_{2}=5$*,* ${y}_{1}=1$, *X*_*n*_ is the average of the parameter *X* across all basins, *X*_max_ is the maximum value of parameter *X*, and *X*_min_ is the minimum value of parameter *X*. All normalized parameters within the basin are added to generate the total risk associated with the basins.

#### Correlation and multicollinearity

In flood hazard mapping, identifying the presence of linear dependency between flood conditioning factors is essential, and multicollinearity and Pearson’s correlation stand out as crucial steps for this purpose (Ruidas et al. 2022b). When one variable is highly correlated with others and can be accurately predicted from them, multicollinearity exists (Al-Juaidi et al. 2018). High correlation and multicollinearity can have a negative impact on the interpretability and performance of the models (Deroliya et al. 2022). To detect linear dependency, the conventional framework in flood hazard studies employs Pearson’s correlation coefficient (*r*) (Mazumder et al. 2022), variance inflation factor (VIF) (Towfiqul Islam et al. 2021), and tolerance (TLT) (Liao and Valliant 2012). In this study, both *r* and VIF values are used to eliminate linear dependency, with a threshold value of 0.7 for Pearson’s correlation (Rahman et al. 2021). VIF values should be smaller than 10 for acceptable results and smaller than five preferably for better results (Tehrany et al. 2019; Ruidas et al. 2022a). The tests are performed on each variable utilized in flood hazard mapping by treating it as the dependent variable and all other variables as independent variables.

#### Frequency ratio method (FR)

The FR is a bivariate statistical model revolving around understanding the probabilistic relationship between dependent and independent variables (LAXTON 1996). The method is commonly applied in flood studies because of its easy implementation and comprehension, and its adaptability with multiple spatial datasets with different classifications (Samanta et al. 2018). The method is applied by dividing the 154 flood locations in Cyprus into two groups, 70% used for training and 30% for testing. The FR is measured using the training datasets and flood hazard parameters as the ratio of the area where flood occurrence may be observed to the total study area. The higher the FR the stronger the correlations, typically, FR values above 1 indicate strong correlations and higher flood hazard, FR values under 1 show weaker correlations (El-Magd 2019). FR values and flood hazard index (FHI) are calculated with the following equations (Aditian et al. 2018):

10 $${\text{FR}}= \frac{({N}_{ij}/{A}_{ij})}{({N}_{r}/{A}_{r})}$$

11 $${\text{FHI}} = \sum_{i=1}^{N}FR$$

where ${N}_{ji}$ is the area of floods in the within class *j* of the parameter *i*, ${A}_{ji}$ is the area of class *j* in parameter *i*, and ${N}_{r}$ and ${A}_{r}$ are the total areas of floods and study area, respectively.

#### FR Shannon’s entropy method (FR-SE)

The FR-SE is common in flood hazard identification studies (Cabrera and Lee 2020; Arora et al. 2021). The entropy of flood events indicates the influence of flood hazard parameters on flood occurrence (Wang et al. 2021). The method is used to calculate relative weights for hazard inducing parameters using the following equations (Liuzzo et al. 2019):

12 $${P}_{ij}= \frac{{{\text{FR}}}_{ij}}{\sum_{j=1}^{{S}_{j}}{{\text{FR}}}_{ij}}$$

where ${P}_{ij}$ is the probability density and FR are the frequency ratio values for class $j$ of factor $i$. ${S}_{j}$ is the total number of classes. The entropy values of ${H}_{{\text{j}}}$ and ${H}_{j{\text{max}}}$, the information coefficient of ${I}_{j}$, and the factor weights are computed as

13 $${{\text{H}}}_{i} = -\sum_{{\text{i}}=1}^{{{\text{S}}}_{{\text{j}}}}{{\text{P}}}_{{\text{ij}}}{{\text{log}}}_{2}{{\text{P}}}_{{\text{ij}}}$$

14 $${{\text{H}}}_{i{\text{max}}}= {{\text{log}}}_{2}{{\text{S}}}_{{\text{j}}}$$

15 $${{\text{I}}}_{i}= \frac{{{\text{H}}}_{i{\text{max}}}-{{\text{H}}}_{i} }{{{\text{H}}}_{i{\text{max}}}}$$

16 $${{\text{P}}}_{i}= \frac{1}{{{\text{S}}}_{{\text{j}}}} \sum_{{\text{i}}=1}^{{{\text{S}}}_{{\text{j}}}}{{\text{P}}}_{{\text{ij}}}$$

17 $${{\text{W}}}_{i}={{\text{I}}}_{i} {{\text{P}}}_{i}$$

The final flood hazard index is then obtained (Bednarik et al. 2010):

18 $${\text{FHI}} = \sum_{i=1}^{n}\frac{Z}{{m}_{i}}\times C\times {{\text{W}}}_{i}$$

where $n$ is the total number of flood conditioning factors, $Z$ is the highest number of classes across all conditioning factors, ${m}_{i}$ is the number of classes of factor $i$, and $C$ is the reclassified value of the factor.

#### Fuzzy analytical hierarchy process technique (F-AHP)

The AHP is widely acknowledged as the most frequently used MCDA method in the flood risk literature (Mudashiru et al. 2021). This method is based on the expert’s pairwise comparison of predictive parameters to determine their relative weights (Younes et al. 2022). The popularity of AHP is due to its hierarchical structure, which enhances the model’s comprehensibility, and its compatibility with consistency analysis (Ekmekcioğlu et al. 2022). However, a major limitation of this method is the subjective nature of human reasoning when providing judgments, and the complexity and uncertainty inherent in real-world problems (Zou et al. 2013; Younes et al. 2022). As a solution to address this limitation, F-AHP is proposed in the literature, which converts pairwise comparisons into fuzzy numbers to address single-number uncertainties (Vilasan and Kapse 2022). F-AHP is used in the study to evaluate its effectiveness with flood vulnerability and hazard, F-AHP is applied in this study, using the following methodology (Chandio et al. 2013):

1. Define the problem and decompose it into a hierarchical structure including the goal, criteria, and subcriteria, as well as the alternatives.
2. Provide a questionnaire to the experts to provide the relative comparison between the input parameters according to the Saaty scale (1–9) (Saaty 1980). Convert the standard Saaty numbers to the triangular fuzzy scale in triangular F-AHP case using Table 3.

**Table 3:** Saaty and triangular fuzzy scales

Linguistic explanation	Saaty number	Reciprocal	Triangular fuzzy scale	Reciprocal triangular fuzzy scale
Equally important	1	1	(1,1,1)	(1,1,1)
Intermediate between 1 and 3	2	1/2	(1,2,3)	(1/3, 1/2, 1)
Moderately important	3	1/3	(1,3,5)	(1/4, 1/3, 1/2)
Intermediate	4	1/4	(3,4,5)	(1/5, 1/4, 1/3)
Strongly important	5	1/5	(3,5,7)	(1/6, 1/5, 1/4)
Intermediate	6	1/6	(5,6,7)	(1/7, 1/6, 1/5)
Very strongly important	7	1/7	(6,7,8)	(1/8, 1/7, 1/6)
Intermediate	8	1/8	(7,8,9)	(1/9, 1/8, 1/7)
Extremely important	9	1/9	(9,9,9)	(1/9, 1/9, 1/9)

3) Consistency of the expert weights is tested using the consistency ratio (Peng and Zhang 2022):
  19 $${\text{CR}}=\frac{{\text{CI}}}{{\text{RI}}}$$
  20 $${\text{CI}}=\frac{1}{N-1}({\lambda }_{{\text{max}}}-n)$$

where ${\text{CR}}$ is random consistency ratio, ${\text{CI}}$ is the general consistency index, ${\text{RI}}$ is the average random consistency index obtained from Table 4, $N$ is the size of the comparison matrix, and ${\lambda }_{{\text{max}}}$ is the largest eigenvalue. Consistent judgments have an accepted threshold value for ${\text{CR}}$, which is 10%.

**Table 4:** Average random consistency index values

Order	1	2	3	4	5	6	7	8	9
RI	0	0	0.58	0.9	1.12	1.24	1.32	1.41	1.45

4) Steps 2 and 3 are completed for each expert opinion taken, and the expert comparison matrices are synthesized together for F-AHP using the geometric mean method (Liu et al. 2020):where ${\widetilde{C}}_{ij}^{(t)}= ({l}_{ij}^{(t)}, {m}_{ij}^{(t)}, {h}_{ij}^{(t)})$ is the triangular fuzzy number showing relative importance of parameter ${C}_{i}$ relative to ${C}_{j}$ and ${\widetilde{C}}_{ij}= ({l}_{ij}, {m}_{ij}, {h}_{ij})$ is the synthesized importance of ${C}_{i}$ relative to ${C}_{j}$.
  21 $$\begin{array}{c}{\widetilde{C}}_{ij}= ({l}_{ij}, {m}_{ij}, {h}_{ij}) = {({\prod }_{t=1}^{N}{\widetilde{C}}_{ij}^{\left(t\right)})}^\frac{1}{N} = {({\widetilde{C}}_{ij}^{\left(1\right)} \otimes {\widetilde{C}}_{ij}^{\left(2\right)} ... \otimes {\widetilde{C}}_{ij}^{\left(3\right)})}^\frac{1}{N}1\\ = \left[{({\prod }_{t=1}^{N}{l}_{ij}^{\left(t\right)})}^\frac{1}{N}, {({\prod }_{t=1}^{N}{m}_{ij}^{\left(t\right)})}^\frac{1}{N}, {({\prod }_{t=1}^{N}{h}_{ij}^{\left(t\right)})}^\frac{1}{N}\right]\end{array}$$

5) The relative weights are generated for F-AHP using geometric mean method with the following equations (Younes et al. 2022):
  22 $${\widetilde{W}}_{i}=\frac{{\widetilde{C}}_{i}}{{\sum }_{j=1}^{N}{\widetilde{C}}_{i}}$$
  23 $${\widetilde{C}}_{i}={({\widetilde{C}}_{i1}*{\widetilde{C}}_{i2} .. .*{\widetilde{C}}_{in})}^\frac{1}{N}$$
6) F-AHP weights are defuzzified using center of area method (Tella and Balogun 2020) as $W=(l+m+h)/3$.
7) F-AHP weights are normalized to have the sum of 1.
8) Flood hazard index (FHI) or flood vulnerability index (FVI) is generated:
  24 $$\mathrm{FHI }({\text{FVI}})={\sum }_{i=1}^{N}{W}_{i}*{R}_{i}$$

where ${W}_{i}$ is the weight of hazard or vulnerability parameter $i$ obtained from F-AHP, ${R}_{i}$ is the reclassed value of parameter $i$, and $N$ is the total number of flood hazard or vulnerability parameters.

#### Receiver operating characteristic curve (ROC)

Validation of the performance of the models is important to know the capability of the models and draw a comparison between them. Receiver operator characteristics curve (ROC) and area under the curve (AUC) are widely used in flood risk studies (Luu et al. 2019; Feizizadeh et al. 2021) to assess model performance. The curve is plotted using the specificity on the *x*-axis (false positive rate) against the sensitivity on the *y*-axis (true positive rate). Then, AUC is calculated to demonstrate the predictive likelihood between a point’s existence and absence in the model (Norallahi and Seyed Kaboli 2021). AUC values can range from 0 to 1, where higher values show better model performance. Typically, AUC values ranging from 0.5 to 0.6, 0.6 to 0.7, 0.7 to 0.8, 0.8 to 0.9, and 0.9 to 1 show poor, average, good, very good, and excellent performance, respectively (Yesilnacar 2005). ROC and AUC were generated using the tools available in ArcGIS (Brown et al. 2017). For the models, ROC and AUC were constructed and tested with 70% training points and 30% testing points.

## Results

### Morphometric risk assessment

**Fig. 5:**
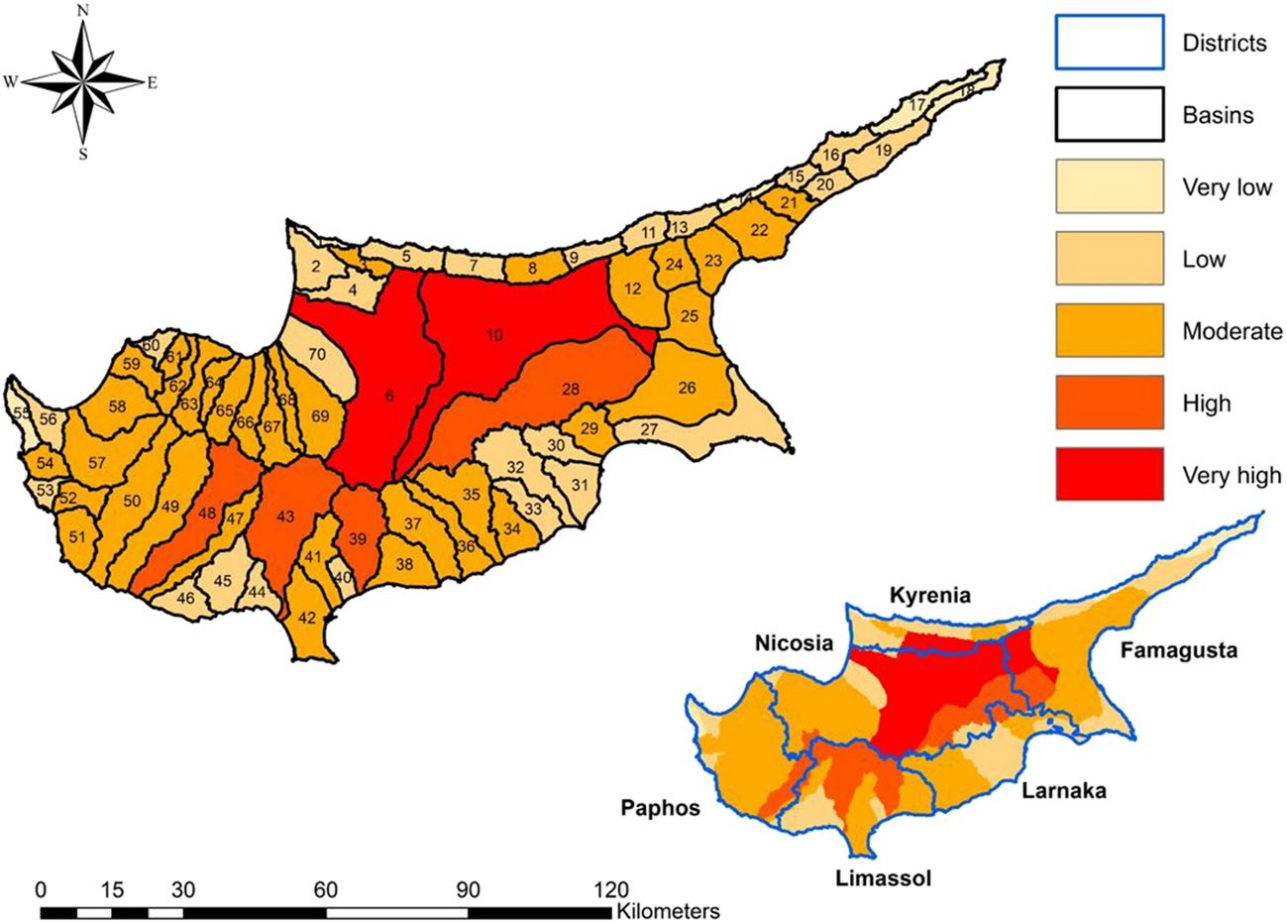
General morphometric risk map

The morphometric parameters for the basins and streams were calculated using ArcGIS and are available for detailed examination in the supplementary document (S.M 2 and S.M 3). The general morphometric risk map (Fig. 5) is generated by considering the scale, shape, topographic, and drainage network parameters across different categories. The results indicated that out of the 70 basins studied, five of them were classified as very low flood risk, 24 as low risk, 35 as mediocre risk, four as high risk, and two as very high risk. Basins 6 and 10, which contain the Serrachis and Pedieos rivers on the island (the largest rivers on the island), exhibited the highest risk among the basins. Among the 108 recorded flood events, 48 of them occurred within the basins classified as very high risk. The high-risk basins encompass a total area of 1604 km2 and have a population of approximately 400,000 people, with a significant population concentration representing around one-third of the total population of the island. Moreover, basins 28, 39, 43, and 48, characterized by high flood potential, recorded 21 historical floods. They collectively cover an area of 1400 km^2^ and house a population of nearly 60,000 people. Therefore, it is crucial to analyze the districts located within highly prone zones to prioritize flood mitigation efforts in these basins (Adnan et al. 2019). The districts of Nicosia, Kyrenia, Famagusta, Larnaka, Limassol, and Paphos have areas of 1685 km^2^, 165 km^2^, 344 km^2^, 49 km^2^, 603 km^2^, and 158 km^2^, respectively, within the high and very high flood risk zones. Consequently, based on the morphometric analysis, the districts of Nicosia and Limassol should be given priority in flood mitigation efforts.

### Correlation and multicollinearity

**Fig. 6:**
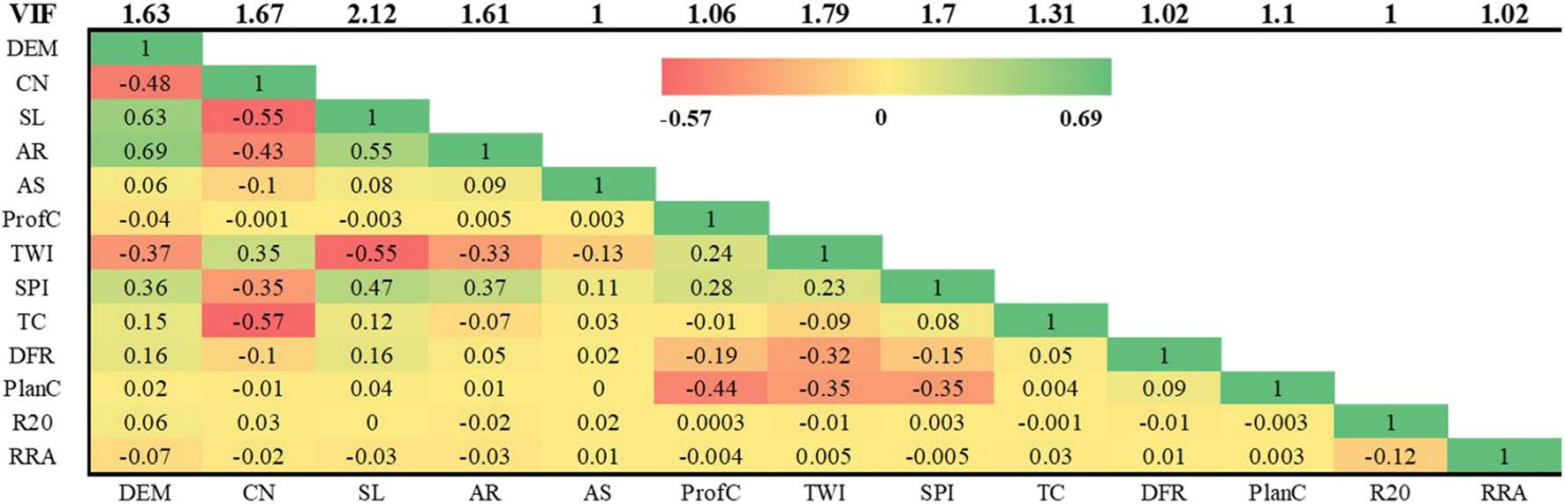
Multicollinearity and correlation diagnostics

Figure 6 presents the findings obtained from the correlation analysis and multicollinearity test. These assessments were carried out on a grid scale to investigate the linear interdependencies among the parameters. The results demonstrated that the VIF values ranged from 1 to 2.12, while the correlation coefficients exhibited variations within the range of − 0.57 to 0.69. Particularly, it should be noted that all correlation coefficients between the parameters were between − 0.7 and 0.7, indicating a modest level of correlation. Additionally, the calculated VIF values were considerably lower than 5. As a result, it was determined that all flood-causing elements could be included in the analysis.

### Flood hazard mapping

#### FR application

The FR analysis was conducted by partitioning the flood points into two groups: a 70% training set and a 30% validation set. The flood hazard factors were subsequently reclassified based on their impact on flood occurrence, as outlined in Table 5. Spatial analysis was performed using GIS to calculate the areal percent histogram of the factor classes and determine the percentage of flood training points within each factor class. Then, the FR values corresponding to the classes were calculated. Herein, the FR values serve as indicators of the correlation between the factor class and flood occurrence, with values exceeding one denoting a strong correlation. Detailed results for FR method are provided in S.M 4. Table 5 denotes the final calculated FR values.

**Table 5:** Calculated FR values

Factor	Reclassified factor	Reclassified value	FR
MR (G)	Very low risk Low risk Moderate risk High risk Very high risk	1 2 3 4 5	0 0.82 0.85 **1.17** **1.6**
RRA (H)	0–0.05 0.05–0.2 0.2–0.39 0.39–0.7 0.7–1.86 1.86–3.17 3.17–20.02	7 6 5 4 3 2 1	0.52 0.34 **1.34** 0.8 **1.63** **1.14** **1.38**
CN (H)	70–74 74–79 79–83 83–86 86–89 89–95 95–100	1 2 3 4 5 6 7	0.12 0.58 **3.02** 0.61 0.5 0.58 0
AS (T)	Flat North Northeast East Southeast South Southwest West Northwest North	8 5 9 10 4 6 7 3 1 2	**1.26** **1.12** **1.32** **1.34** **1.02** **1.17** **1.23** 0.7 0.24 0.55
TWI (H)	2.34–5.36 5.36–6.78 6.78–8.47 8.47–10.42 10.42–12.64 12.64–15.74 15.74–24.97	1 2 3 4 5 6 7	0.4 **1.34** **1.16** **1.36** 0.64 0.63 1.08
SPI (H)	− 3.69 to − 0.50 − 0.50 to 1.1 1.1–2.54 2.54–3.84 3.84–5.51 5.51–8.02 8.02–15.69	1 2 3 4 5 6 7	**1.31** **1.66** **1.42** 0.27 0.61 0.19 0.58
TC (H)	0.05–0.39 0.39–0.68 0.68–1.05 1.05–1.55 1.55–2.28 2.28–3.46 3.46–6.77	7 6 5 4 3 2 1	**1.93** 0.18 0.48 0 0 0 0
ProfC (T)	Upwardly convex Linear Upwardly concave	1 2 3	0.93 0.89 **1.09**
PlanC (T)	sideward concave linear sideward convex	3 2 1	0.85 **1.26** **1.02**
DEM (T)	0–120 120–266 266–438 438–641 641–886 886–1223 1223–1954	7 6 5 4 3 2 1	**1.12** **1.12** 0.73 **1.14** 0.49 0.86 0.81
Sl (T)	0–1.22 1.22–2.56 2.56–4.85 4.85–8.70 8.70–14.68 14.68–22.67 22.67–71.36	7 6 5 4 3 2 1	**3.75** **1.85** 0.53 0.26 0.39 0.07 0
AR (M)	233–357 357–436 436–510 510–601 601–699 699–812 812–971	1 2 3 4 5 6 7	**1.96** **1.40** 0.89 **1.25** 0.32 0.53 0.89
DFR (H)	0–48.18 48.18–103.778 103.778–159.368 159.368–211.25 211.25–266.84 266.84–337.25 337.25–945.05	7 6 5 4 3 2 1	**1.32** **1.00** 0.73 **1.41** 0.44 0.45 0.00
R20 (M)	124–212 212–263 263–307 307–354 354–402 402–467 467–579	1 2 3 4 5 6 7	**2.28** **1.25** **1.03** 0.99 0.97 0.37 0.43

Bold indicates significant FR (> 1)

As seen in Table 5, in the case of MR, the FR values demonstrated a direct correlation with flood occurrence, with high and very high classes exhibiting FR values of 1.17 and 1.6, respectively. RRA exhibited significant FR values in four classes, with the highest value of 1.63 observed within the 0.7 to 1.86 range. CN values ranging from 79 to 83 demonstrated a very high FR value of 3.02, indicating that the majority of floods in the study area occur within this range. AS exhibited significant FR values in seven classes, with the eastern class being the most prominent, characterized by an FR of 1.34. TWI and SPI displayed prominent FR values in the ranges of 8.47 to 10.42 and − 0.50 to 1.1, respectively, with corresponding FR values of 1.32 and 1.66.

TC demonstrated a negative relationship with flood occurrence, with the highest FR value of 1.93 observed in the lowest TC zones. ProfC and PlanC indicated that the majority of flood occurrences transpire on upwardly concave and sideward linear surfaces, respectively. DEM and SL exhibited a negative relationship with flood occurrence, with the highest FR values of 1.12 and 1.14 observed in lower elevation zones (0 to 266 m) and lower slope zones (0 to 1.22), respectively. AR and R20 displayed higher FR values with lower AR and R20 values, which can be attributed to the concentration of recorded floods in the central region of the Nicosia district, characterized by relatively lower total rainfall amounts throughout the year. Finally, DFR demonstrated significant FR values in three ranges: 0 to 48.18, 48.18 to 103.78, and 159.37 to 211.25, with corresponding FR values of 1.32, 1, and 1.41, respectively.

#### FR-SE application

The FR values obtained from the frequency ratio analysis were utilized in calculating the weights through FR-SE method. The FR values were calculated to determine the probability densities and entropy coefficients. These values were then used to compute the entropy indices $\left({H}_{j}\mathrm{\;and}\;{H}_{jmax}\right)$, the information coefficient $\left({I}_{j}\right)$, as well as the weights $\left({W}_{j}\right)$ and normalized weights $\left({W}_{j}\times Z/{M}_{i}\right)$. S.M 5 presents the thorough calculations for the FR-SE method. Table 6 shows the normalized weights calculated by FR-SE method.

**Table 6:** The F-AHP and FR-SE weights

Factor	F-AHP weights	FR-SE weights
MR (G)	0.03	0.13
RRA (H)	0.02	0.04
CN (H)	0.12	0.15
AS (T)	0.02	0.02
TWI (H)	0.08	0.02
SPI (H)	0.07	0.06
TC (H)	0.23	0.15
ProfC (T)	0.04	0.01
PlanC (T)	0.03	0.02
DEM (T)	0.08	0.02
Sl (T)	0.17	0.24
AR (M)	0.02	0.04
DFR (H)	0.07	0.06
R20 (M)	0.02	0.05

Among the factors investigated, MR, CN, SL, and TC exhibited the highest weights, measuring 13%, 15%, 24%, and 15%, respectively. Collectively, these factors accounted for 67% of the total weights, with SL being identified as the most influential factor. The second set of parameters, including RRA, AR, SPI, DFR, and R20, displayed moderate weights of 4%, 4%, 6%, 5%, and 5%, respectively. The resulting flood hazard map was primarily controlled by these two groups of parameters. The third group, consisting of DEM, ProfC, AS, TWI, and PlanC, exerted the least influence on the resulting map, with weights ranging between 1 and 2%. In total, this group contributed 8% of the overall weight distribution.

#### F-AHP factor reclassification and weight calculation

The initial step in obtaining the weights for the flood hazard map involved the application of F-AHP method. The experts in the fields of water resources and hydraulics were engaged to complete a pairwise comparison matrix, which was prepared using the AHP online system (Goepel 2018). In order for the results to be considered acceptable, the consistency ratio (CR) of the matrix needed to be below 10%. The calculated CR was determined to be 9.1%, indicating the consistency and reliability of the results. Subsequently, the pairwise comparison matrix was fuzzified using triangular fuzzy membership conversions. The original pairwise matrix and fuzzified matrix can be observed in the in the S.M 6. The fuzzified matrix was then employed to calculate geometric mean values, fuzzy weights, defuzzified weights, and normalized weights, provided in detail in S.M 7. Table 6 provides final weights calculated with F-AHP method. Analysis of the weights revealed that CN, SL, and TC exhibited the highest levels of significance, with respective weights of 0.12, 0.17, and 0.23. Following these factors, SPI, DFR, DEM, and TWI demonstrated moderate weights of 0.07, 0.07, 0.08, and 0.08, respectively. MR and PlanC had notably low weights of 0.03, resulting in minimal influence on the generated flood hazard map. Lastly, RRA, AR, AS, and R20 were identified as the least significant parameters within the study area, characterized by a weight of 0.02. Finally, a spatial overlay was conducted by multiplying the reclassified factors with the normalized weights, resulting in the generation of the flood hazard map. A detailed results about the weights is given in S.M 8.

#### Validation and assessment of the methods

**Fig. 7:**
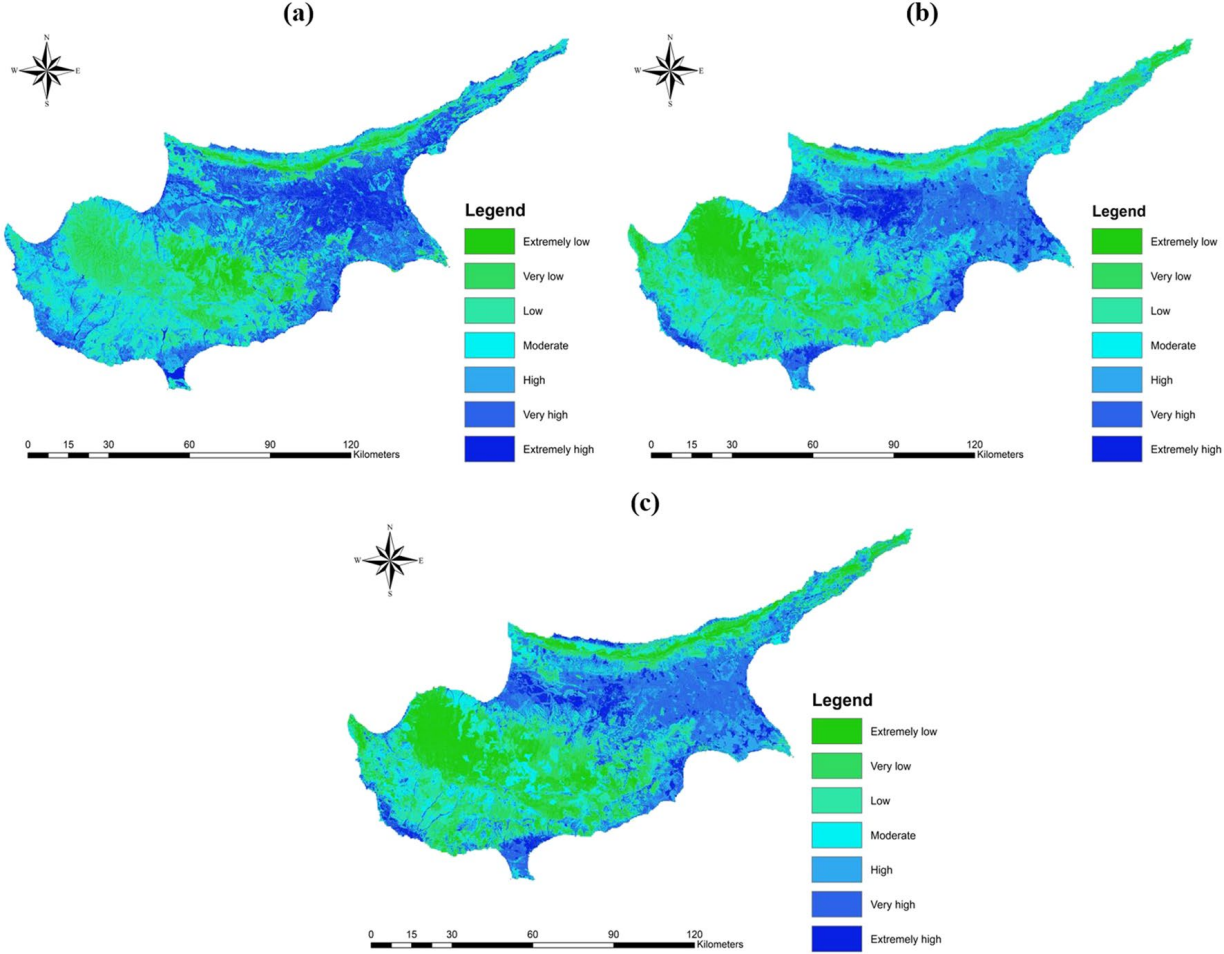
Flood hazard maps generated by employing **a** F-AHP, **b** FR, and **c** FR-SE

**Fig. 8:**
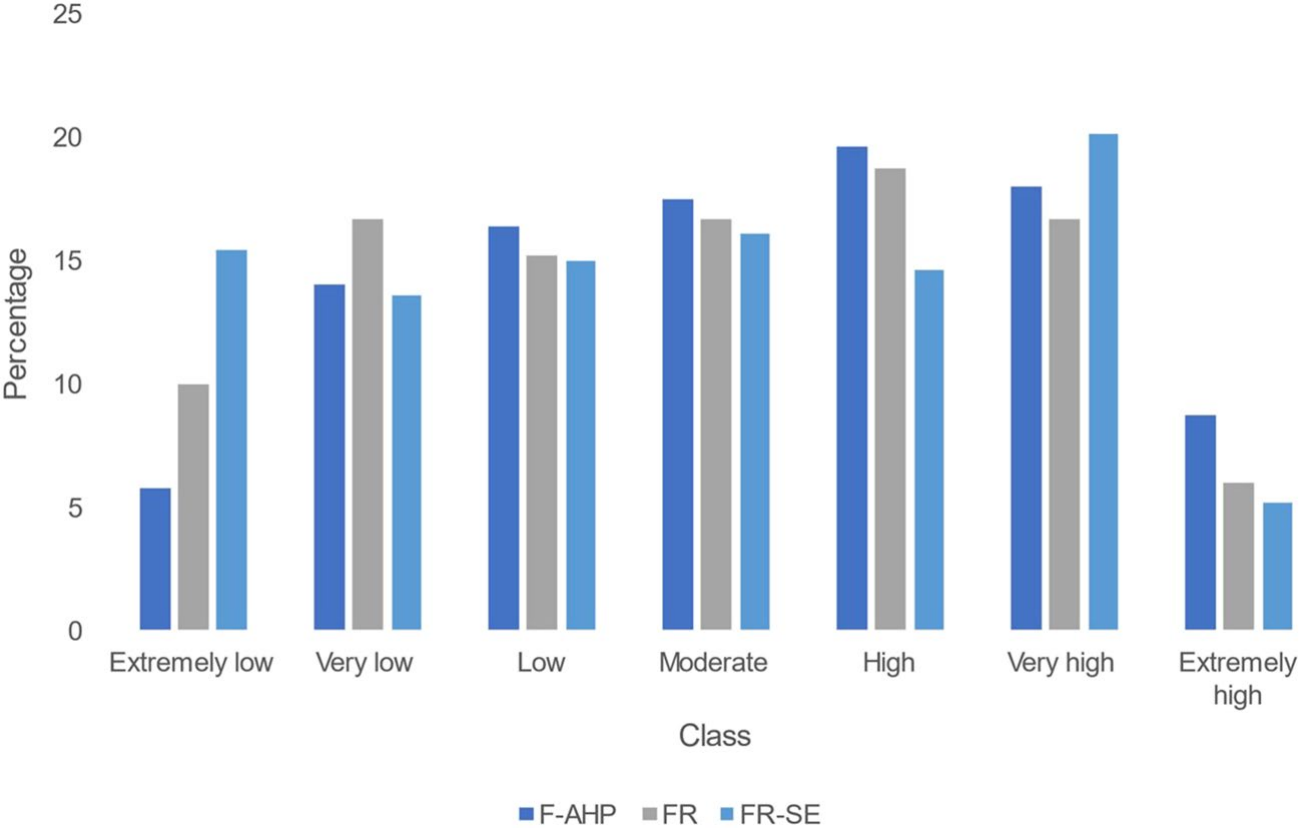
Predicted area wise comparison between the methods

The flood hazard hazard maps were generated using overlay analysis in GIS for the three methods. Figure 7 displays the resulting flood maps for the entire island. The maps were reclassified into seven classes using natural breaks, ranging from extremely low to extremely high hazard potential. The maps exhibited similar patterns, indicating lower risks in mountainous and high elevation regions, while flat areas and cities showed higher hazard potential. However, FR and FR-SE methods visually outlined higher risks in cities compared to the F-AHP method, which predicted them as moderate. Figure 8 provides an overview of the distribution of flood classes predicted by the methods in terms of area percentages. The FR-SE method exhibited the highest coverage in the extremely low to low flood class, encompassing 44% of the study area. Among the moderate flood class, the F-AHP method had the highest percentage with 17.5%, followed by 16.7% in FR and 16.1% in FR-SE. In particular, the F-AHP method surpassed the other methods in terms of the high to extremely high flood zones, covering 46.3% of the entire island.

**Fig. 9:**
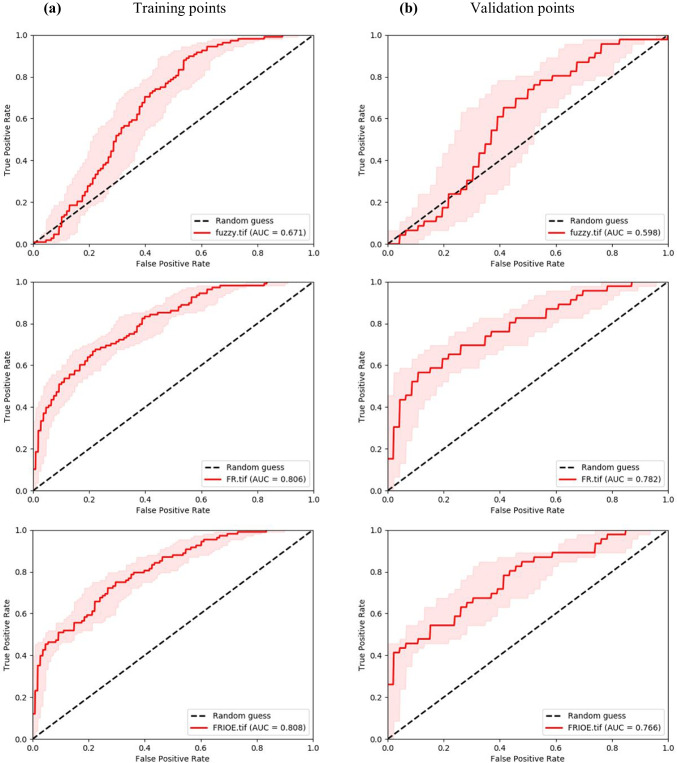
ROC-AUC validation curves: **a** F-AHP, **b** FR, and **c** FR-SE

To determine the best hazard map, ROC curves were employed and generated for both the training and validation points, as depicted in Fig. 9. The curves revealed that the FR model exhibits the highest predictability, with a very good AUC of 0.806 for training points and a good AUC of 0.782 for validation points. On the other hand, the F-AHP method performed the weakest predictive ability among the analyzed methods, with average AUC results of 0.67 and 0.6 for training and validation, respectively. This limitation could be attributed to the inherent subjectivity of the F-AHP method, which relies on the expertise of the experts. Consequently, the FR flood hazard map was selected as the representative map due to its superior predictive performance.

### Flood vulnerability mapping

**Fig. 10:**
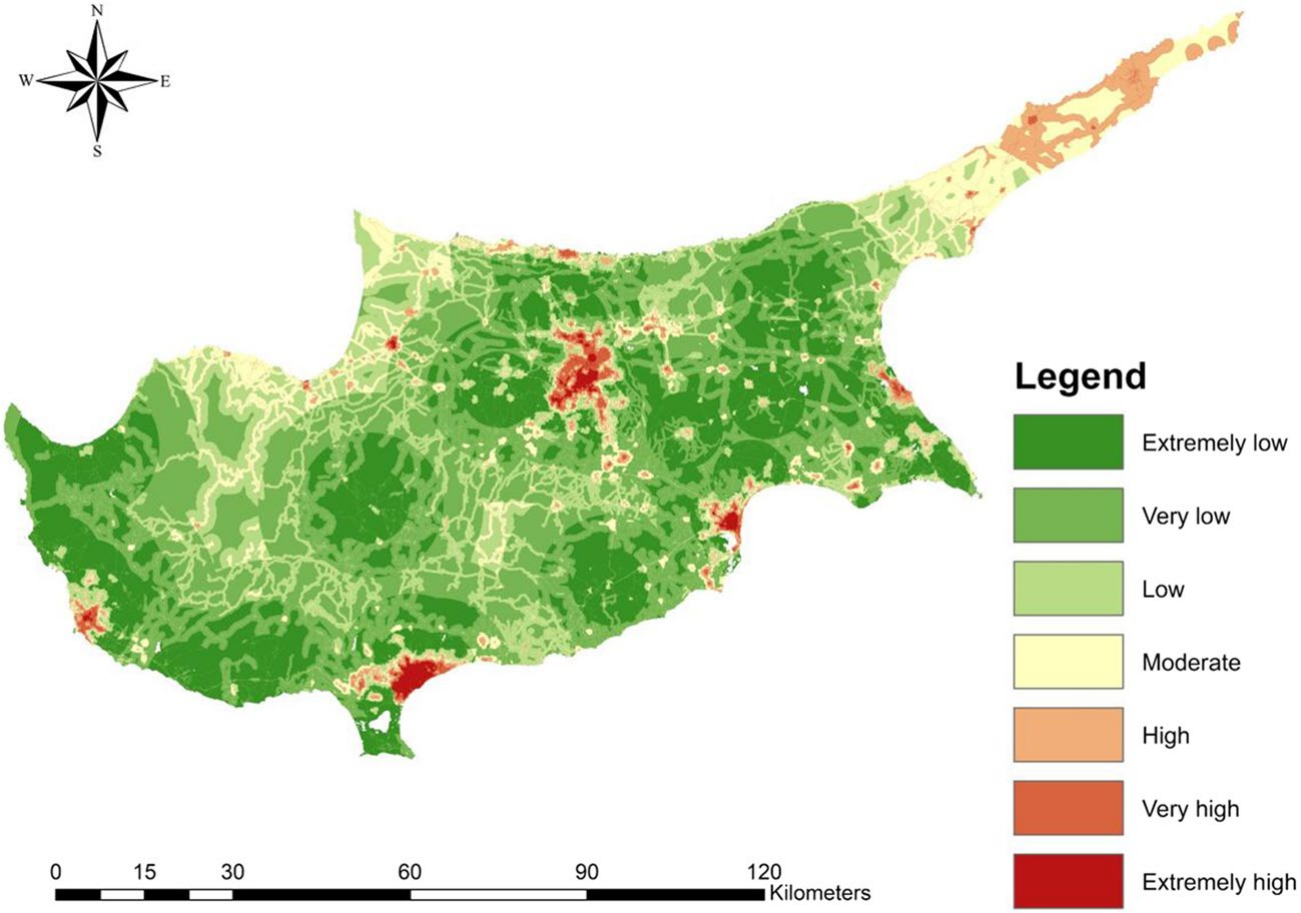
Flood vulnerability index map (FVI)

A flood vulnerability map was generated using the F-AHP method, incorporating expert opinions. The F-AHP method was directly chosen due to the subjective and non-predictive nature of flood vulnerability factors, which do not predict flood occurrence (Eini et al. 2020). The map considered exposure factors such as population (*P*), economic value of buildings (EV), and changes in artificial surfaces (UR), as well as coping capacity factors including road vulnerability index (RVI) and distance from hospitals (DFH). The pairwise matrix, generated by the experts, can be found in the S.M 9, and detailed results of the F-AHP application are provided in S.M 10. The matrix exhibited a consistency ratio of 6.2%, indicating its acceptability. The F-AHP results indicated that *P* holds the highest vulnerability factor weight of 47%, followed by DFH and RVI with weights of 26% and 16%, respectively. EV and UR were found to have less significance, with weights of 7% and 4%, respectively. The final flood vulnerability map (Fig. 10) was generated by multiplying the natural break reclassified factors by their respective weights and further reclassifying the map into seven classes. The map revealed high to extremely high vulnerability classes in regions located within major cities, attributable to the dense population in these areas. This pattern is consistent across different districts. Besides, the Karpass Peninsula in the Northeast exhibited high flood vulnerability despite having a low population, owing to its low coping capacity in terms of road and hospital accessibility. Overall, 82.5% of the study area fell within the extremely low to low flood vulnerability range, while 11.2 fell within the moderate range, and 6.3% fell within the high to very high range.

### Flood risk mapping

**Fig. 11:**
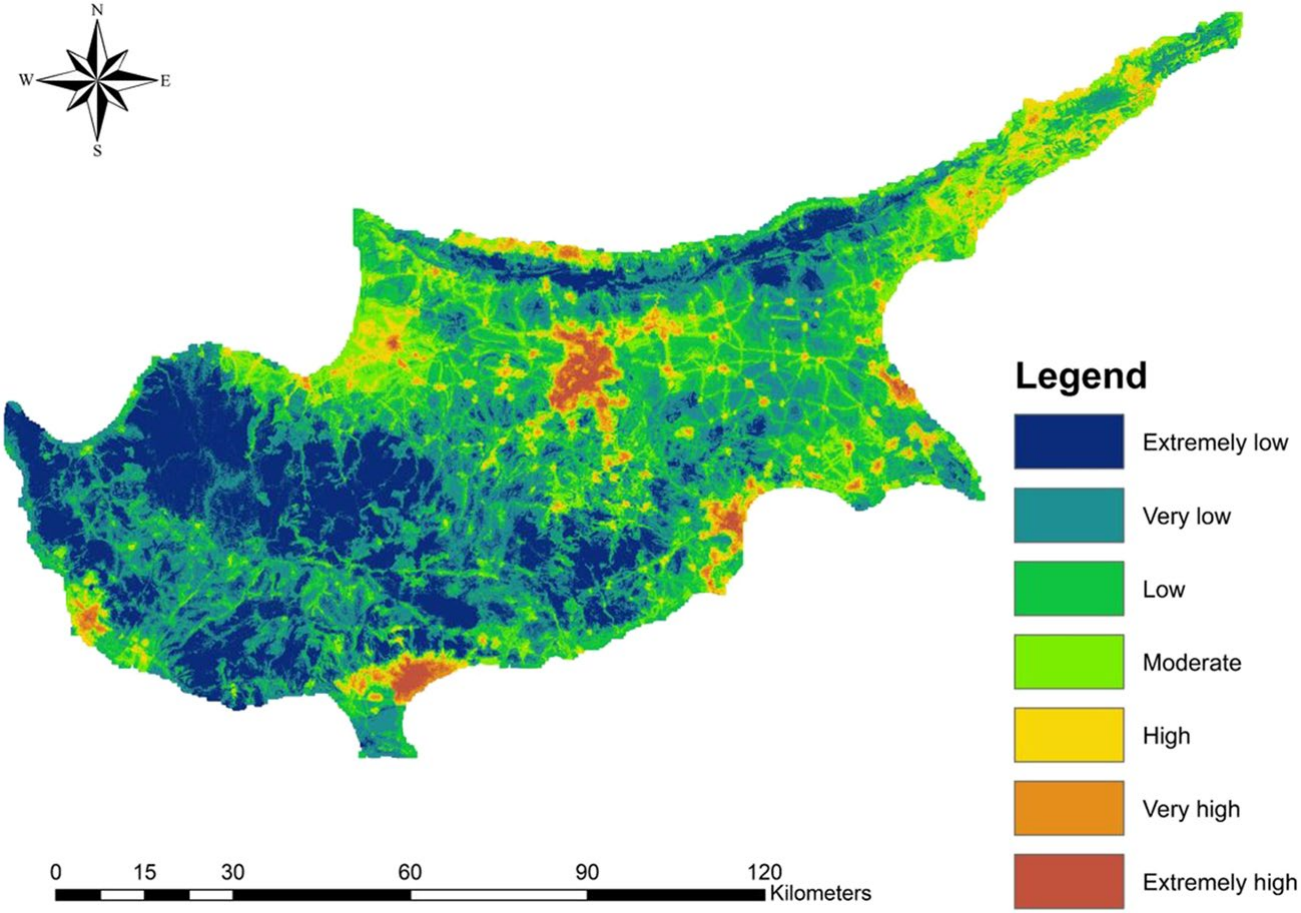
Flood risk map (FRI)

The flood risk index map (Fig. 11) was generated by multiplying the flood hazard map derived from the FR model, which had the highest AUC, with the flood vulnerability map obtained from the F-AHP method. The resulting map was classified into seven classes and provided an integrated view of the combined impact of flood hazard and vulnerability factors. The regions that encompass the central areas of districts and villages exhibited the highest flood risks, ranging from high to extremely high, covering approximately 9% of the study area. These areas were characterized by high population densities, gentler slopes, lower elevations, higher curve numbers, and shorter time of concentration due to urbanization. It should be noted that the flood risks in several zones of the Karpass Peninsula were underestimated, despite the fact that this region poses significant risks. Moderate flood risks covered 13.2% of the study area and were associated with the regions exhibiting moderate hazard and vulnerability properties. The remaining portions of the study area were assigned with extremely low to low flood risks. These areas were predominantly located in the Kyrenia and Troodos Mountain regions, characterized by steep slopes, higher elevations, green land use, and lower population densities. Additionally, non-urbanized agricultural regions situated between cities also had extremely low to low flood risk levels, owing to the relatively low hazard conditions prevalent in these areas.

### Practical application: unveiling the significance and relevance of this study

**Fig. 12:**
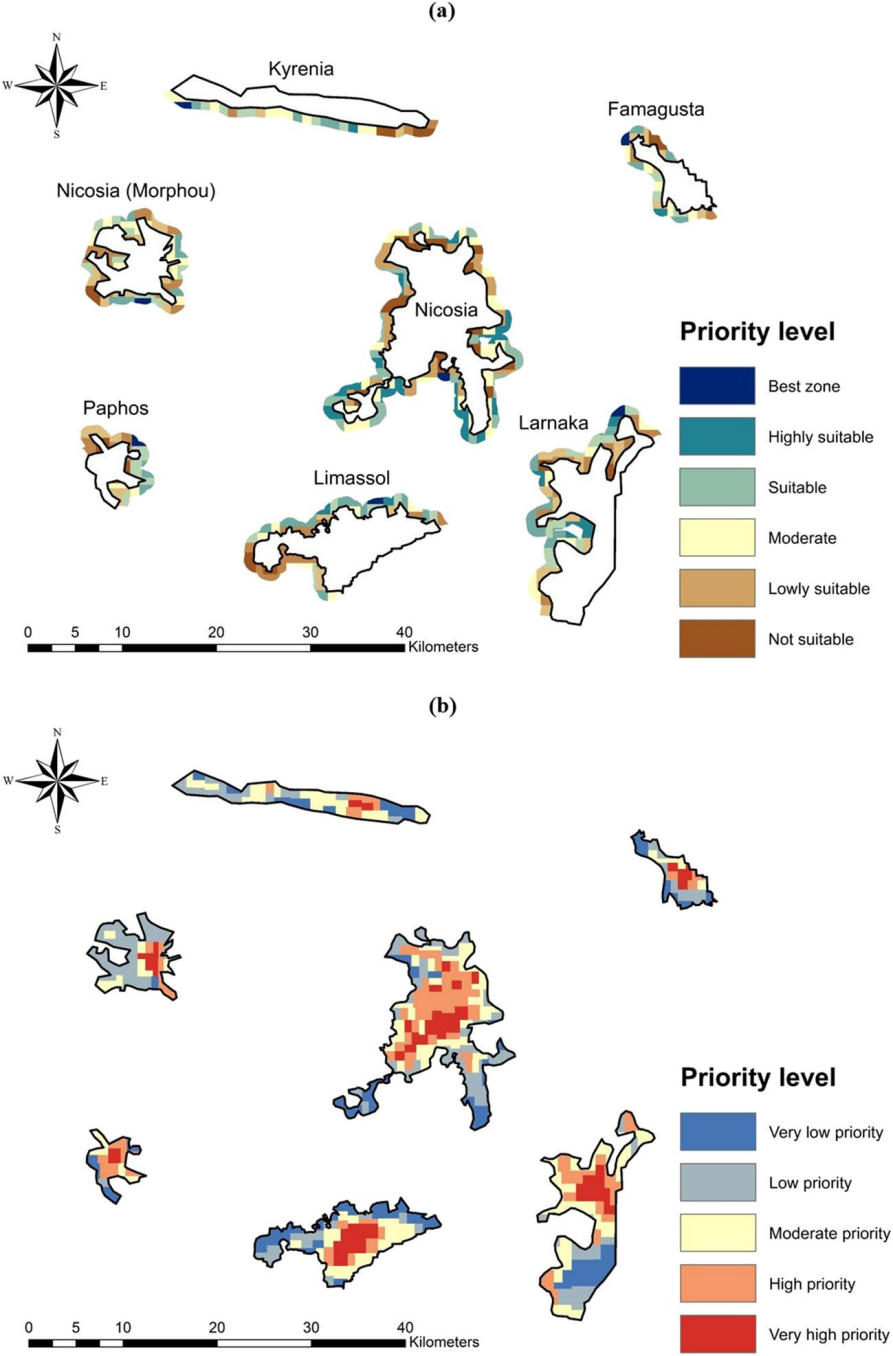
**a** The ranked expansion zones and **b** prioritized zones for flood risk management in the main urban areas of districts of Kyrenia, Nicosia, Famagusta, Paphos, Limassol, and Larnaka

The utilization of a flood risk map can aid in the identification of city expansion regions characterized by lower flood risk. By examining the flood risk levels within cities, it is possible to identify expansion zones with comparatively lower flood risk, thus making them suitable for future urban development. However, it is essential to recognize that even the recommended expansion zones may encounter certain local challenges that require attention. Hence, it is recommended to assign a suitability ranking to all expansion zones, encompassing a range from unsuitable to highly suitable regions. This ranking framework provides valuable guidance to local authorities and city planners in their decision-making processes. In this context, this study employed a certain methodology to rank the potential expansion zones for the districts in Cyprus. Initially, the study assumed that no new cities would be established, and instead, the existing districts characterized by high to extremely high flood risks would undergo expansion. Subsequently, utilizing GIS, these districts were buffered by a user-defined distance of 1 km to delineate the potential expansion zone for each district. To ensure uniform areas of 1 km^2^, the expansion zone buffer for each district was further subdivided into distinct expansion polygons. Then, the average flood risk associated with each expansion polygon was computed. Based on their suitability, the zones were ranked, spanning from regions deemed unsuitable to highly suitable, as illustrated in Fig. 12a. Herein, the expansion polygon exhibiting the lowest flood risk was considered the most suitable expansion zone. Flood risk maps can also be utilized in the context of flood risk management within existing cities. Similar to determining expansion zones, the cities were subdivided into equal areas of 1 km^2^, and the average flood risk corresponding to each zone was computed. The inner zones were then ranked and classified into five flood management priority classes: very high priority, high priority, moderate priority, low and very low priority, as illustrated in Fig. 12b. It is recommended that regions with higher risks should be prioritized for flood management funding and practices. Particularly, as an adaptation to climate change, promoting sustainable water harvesting and management practices, such as rooftop harvesting, permeable pavements, green roofs, and early warning systems could potentially reduce the flood risk in the city, benefiting local communities.

## Discussion

Cyprus over the past decades has continuously been exposed to floods, which caused significant economic and infrastructural damages. Understanding the spatial distribution of flood prone areas, the interaction and significance of flood triggering factors, and the vulnerability after the occurrence of floods is a crucial step in achieving an integrated flood management system. Therefore, an extensive quantitative analysis was conducted in the study area, analyzing the impact of the flood-inducing factors, and examining the performance of three predictive models, namely, F-AHP, FR, and FR-SE models. In addition, flood hazard and vulnerability factors were quantitatively analyzed to demonstrate their significance in generating the flood risk map. The study showed that weights of the factors vary significantly depending upon the employed model, which is aligned with existing literature (Mudashiru et al. 2021). For instance, FR results showed the most influential hazard factors to be AS and RRA in Cyprus. Similar research conducted by Vilasan and Kapse (2022) found slope to be the most significant factor in Ernakulam district of India, while Goumrasa et al. (2021) conducted a study utilizing nine flood triggering factors in Chifa Wadi watershed, Algeria, where they predicted elevation as the most influential hazard factor. Wu et al. (2022) emphasized that dominant hazard factors vary depending upon the studied geographic region. Therefore, flood mitigation proposals should be based on nationally conducted flood assessment endeavors.

Statistical ROC tests were used to test the predictive performance of the model. The results demonstrated that FR has the highest predictive ability, achieving an AUC score of 80%, closely followed by FR-SE with 79%, finally, F-AHP displayed the lowest predictive ability at 60%. These results align with Khosravi et al. (2016), where FR method outperformed AHP and FR-AHP ensemble. Conversely, Hasanuzzaman et al. (2022) and Tariq et al. (2022) reported higher prediction rates in AHP methods. The literature consistently shows FR and FR-SE to possess a similar predictive performance (Arora et al. 2021; Sarkar et al. 2022). AHP methods are subjective in nature, depending upon expert experience without considering historical flood locations, introducing a degree of uncertainty reflected in their poor predictive performance in this study. Therefore, caution should be exercised in the use of these methods, putting in mind careful expert choice, sensitivity analysis, and validation are highly recommended in AHP applications. Another mode of uncertainty stems from errors associated with the spatial representation of the data. Future studies will aim at obtaining higher resolution datasets and utilizing them in flood hazard assessments.

In our study, we identified high flood risks in the main cities of Cyprus, concentrated heavily in the districts of Nicosia, Larnaka, and Limassol. The Nicosia district ranging from the west of the island to the center has the highest area-wise flooding risks. This is attributed to multiple factors, including the low elevations on the island, the flat terrain, and presence of condensed and large mountain stream networks. Additionally, as the capital city of the island, Nicosia has the highest population density, rendering it the most vulnerable. Consequently, adequate city planning, continuous inspection of local flooding problems, and proposal of sustainable solutions are recommended. The western part of the district includes the agricultural subdistrict of Morphou, prone to floods, and containing the largest aquifer on the island. The aquifer has been over exploited over the past few decades due to unsustainable practices. Implementation of water collection practices and diverting it to the aquifer would assist in returning it to a healthy state. Previous research was conducted to map the flood hazard regions on the island. For example, Franci et al. (2016) used AHP to delineate the flood risks in the Yialias river basin encompassing the Agia Varvara, Pera Chorio, and Dali villages, where they uncovered the proneness of the region to flooding, aligning with the outcome of this paper. Further in the same basin, Alexakis et al. (2014) investigated the impact of LULC change on the predicted runoff, uncovering an increase in runoff due to urbanization in the region. The findings of those mentioned studies corroborate the results of this paper, which employed a comprehensive methodological approach to extend the analysis to the whole island.

## Conclusion

Unplanned urban development and climate change are anticipated to have significant impacts on the occurrence of floods. To address this multi-criteria analysis problem, the flood risk zoning map has been proposed as a valuable tool to support local authorities in making informed decisions regarding urban planning, flood control, and response strategies. In this context, the objective of this research is to perform an extensive flood risk analysis on the island of Cyprus, utilizing geographic information systems and remotely sensed datasets, with the aim of mapping and evaluating the collective impacts of hazard and vulnerability factors on flood risk.

In this study, a flood hazard map was developed by computing weights for 14 hazard factors using the methods of F-AHP, FR, and FR-SE. Statistical frequency ratio methods exhibited superior predictability when compared to the subjective F-AHP, with the FR method demonstrating the highest performance. Furthermore, the vulnerability map was generated after computing the weights of five vulnerability factors using F-AHP. After which the maps were utilized to generate the flood risk map, revealing that 9% of the island fell within the high flood risk zones, with the Nicosia district displaying the highest level of risk. These areas in high risk have experienced urbanization with increased population densities, thereby intensifying the associated risks. They exhibited higher curve numbers, leading to reduced infiltration capacity, and shorter time of concentration, highlighting the rapidity at which runoff can be generated by catchments. These factors hold critical importance in formulating evacuation plans, particularly in the context of flash floods. On the other hand, the flood risk map illustrated that the majority of areas with low and extremely low risk were predominantly found in mountainous regions and agricultural areas located between cities.

In conclusion, it is highly recommended that local authorities integrate natural hazard risk maps into future urban planning and resource allocation as an indispensable element of an integrated hazard management system. In particular, there is a compelling need for comprehensive public education initiatives aimed at enhancing public awareness regarding flood risk systems. Adequate allocation of funds toward data collection and analysis is crucial for improving the accuracy and reliability of natural hazard risk assessment. Furthermore, conducting interdisciplinary research encompassing various dimensions, such as psychology, ecology, sociology, economics, climate change, and human behaviors, is strongly encouraged to advance understanding and knowledge in this field.

### Supplementary Information

Below is the link to the electronic supplementary material.

Supplementary file1 (DOCX 3690 KB)

## Data Availability

Data, models and outputs supporting the findings of the study can be provided by the corresponding author upon request.
