# Supplementary material for: A geospatial analysis of flood risk zones in Cyprus: insights from statistical and multi-criteria decision analysis methods
Source: Environ Sci Pollut Res Int. 2024 Apr 26;31(22):32875–900. doi: 10.1007/s11356-024-33391-x (PMC11133077; doi:10.1007/s11356-024-33391-x)
Supplement: Supplementary file 1 — Supplementary file1 (DOCX 3690 KB) [file 11356_2024_33391_MOESM1_ESM.docx]

**Supplementary Material for**

**A Geospatial Analysis of Flood Risk Zones in Cyprus: Insights from Statistical and Multi-Criteria Decision Analysis Methods**

Ma’in Abed Alhakim Naser Ghanem^1*^, Hasan Zaifoglu^2^

^1*^Sustainable Environment and Energy Systems, Middle East Technical University Northern Cyprus Campus: Orta Dogu Teknik Universitesi - Kuzey Kibris Kampusu, Guzelyurt via Mersin 10, 99738 Kalkanli, Türkiye. Email: ghanem.maen.na@gmail.com (*Corresponding author)

^2^Civil Engineering Program, Middle East Technical University Northern Cyprus Campus: Orta Dogu Teknik Universitesi - Kuzey Kibris Kampusu, Guzelyurt via Mersin 10, 99738 Kalkanli, Türkiye. Email: zhasan@metu.edu.tr. <https://orcid.org/0000-0003-2615-5097>

# **1. Curve numbers**

**S.M 1** Corine LULC, Soil texture map, level 2 map and average CN for each LULC class.

| 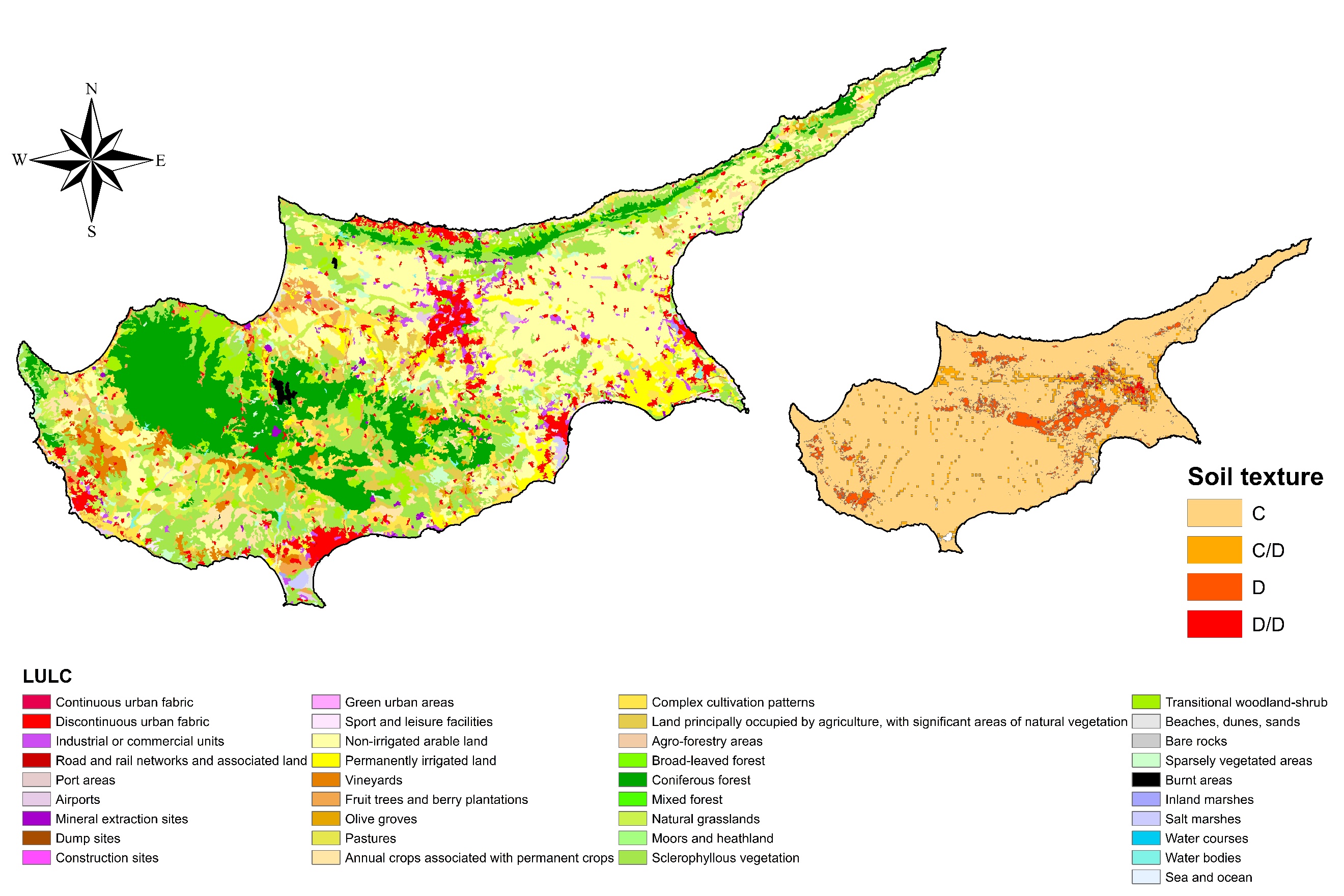 | | |
| --- | --- | --- |
| Landuse type | Average CN | Manning’s n |
| Continuous urban fabric | 90 | 0.012 |
| Discontinuous urban fabric | 86 | 0.012 |
| Industrial or commercial units | 92.5 | 0.012 |
| Road and rail networks and associated land | 98 | 0.016 |
| Port areas | 95 | 0.012 |
| Airports | 94.8 | 0.011 |
| Mineral extraction sites | 92.5 | 0.018 |
| Dump sites | 92.3 | 0.012 |
| Construction sites | 92.5 | 0.012 |
| Green urban areas | 82.3 | 0.018 |
| Sport and leisure facilities | 78.5 | 0.017 |
| Non-irrigated arable land | 89.5 | 0.035 |
| Permanently irrigated land | 88 | 0.035 |
| Vineyards | 84 | 0.018 |
| Fruit trees and berry plantations | 82 | 0.018 |
| Olive groves | 85 | 0.15 |
| Pastures | 82.8 | 0.035 |
| Annual crops associated with permanent crops | 85 | 0.035 |
| Complex cultivation patterns | 85 | 0.035 |
| Land principally occupied by agriculture, with significant areas of natural vegetation | 80.5 | 0.035 |
| Agro-forestry areas | 75.5 | 0.15 |
| Broad-leaved forest | 73.5 | 0.15 |
| Coniferous forest | 74.7 | 0.15 |
| Mixed forest | 70 | 0.15 |
| Natural grasslands | 82.8 | 0.035 |
| Moors and heathland | 75.5 | 0.035 |
| Sclerophyllous vegetation | 77.3 | 0.035 |
| Transitional woodland-shrub | 84.7 | 0.035 |
| Beaches, dunes, sands | 87.3 | 0.02 |
| Bare rocks | 89 | 0.03 |
| Sparsely vegetated areas | 93.3 | 0.018 |
| Burnt areas | 92.5 | 0.018 |
| Inland marshes | 100 | 0.035 |
| Salt marshes | 100 | 0.035 |
| Water courses | 100 | 0.011 |
| Water bodies | 100 | - |
| Sea and ocean | 100 | - |

# **2. Morphometric parameters**

**S.M 2** Spatial distribution of morphometric parameters. Red: Scale parameters, brown: shape parameters, green: topographic parameters, blue: stream network parameters.


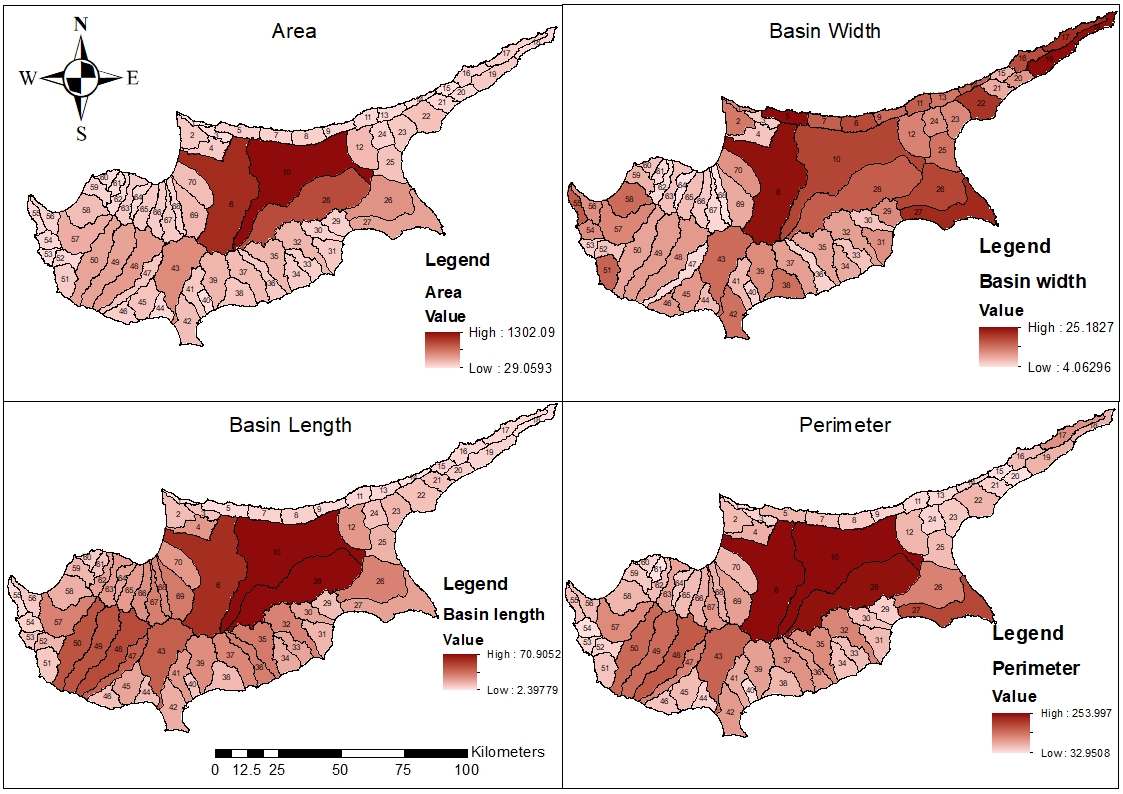


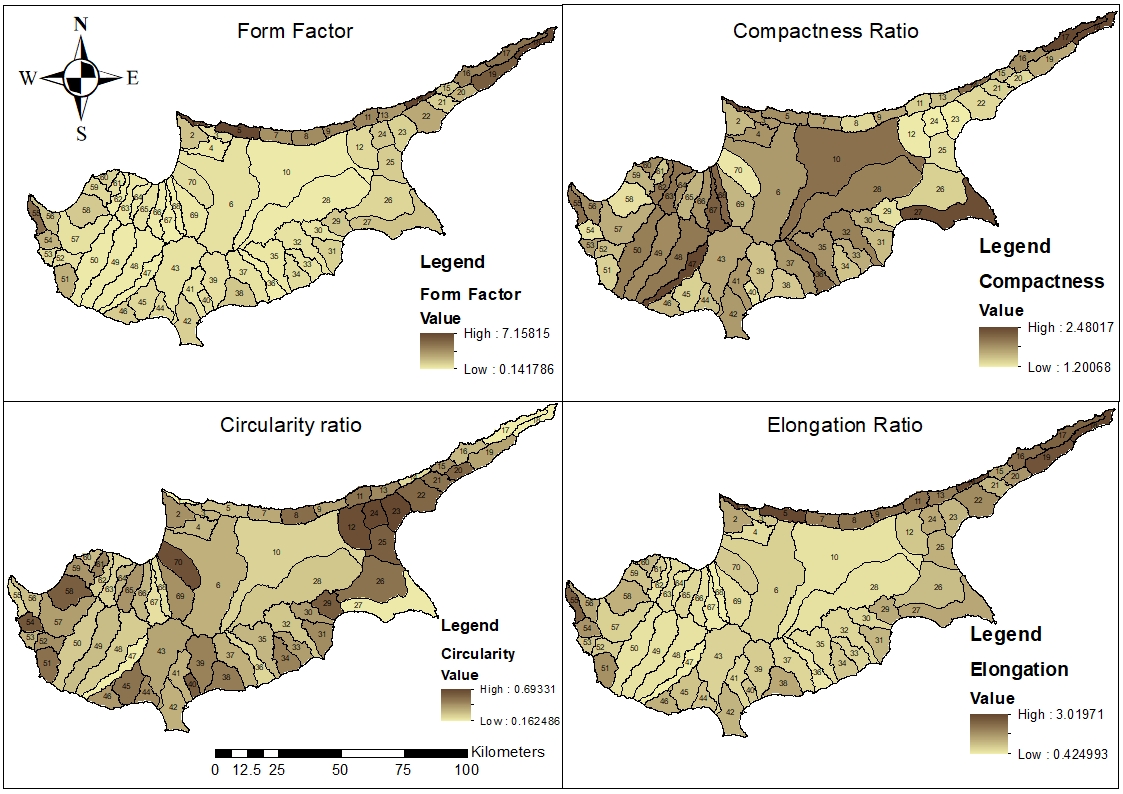

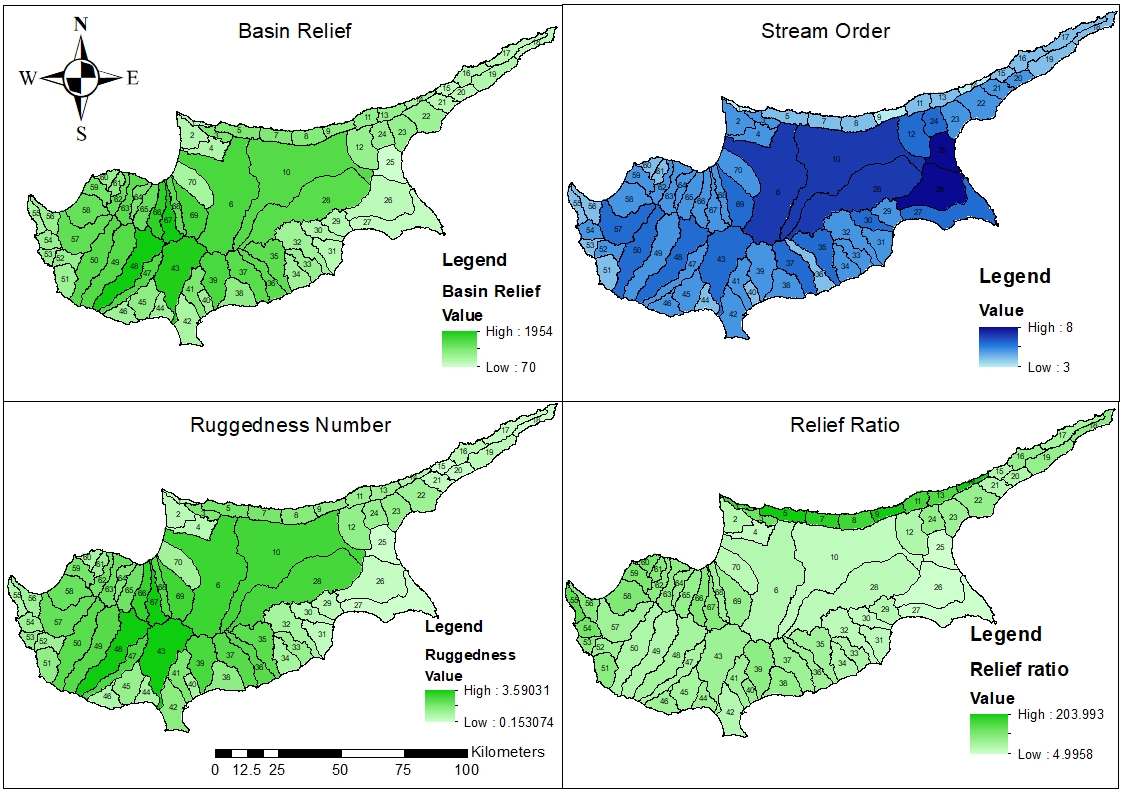

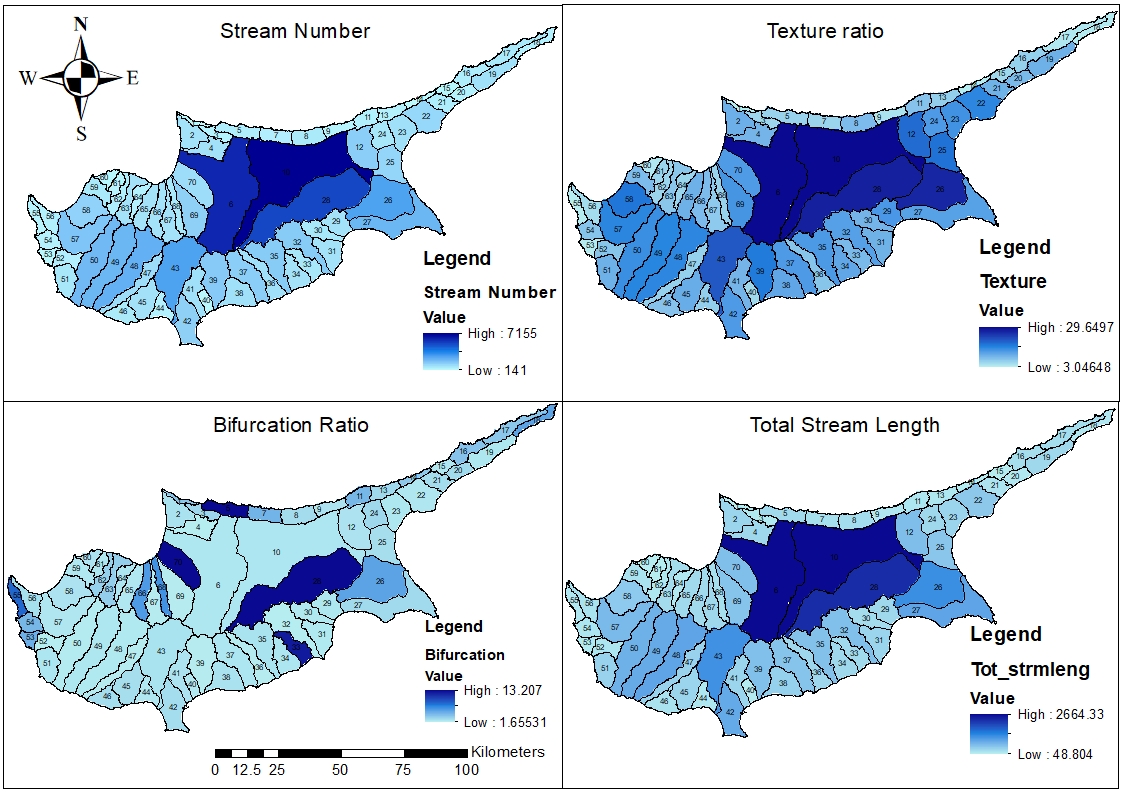

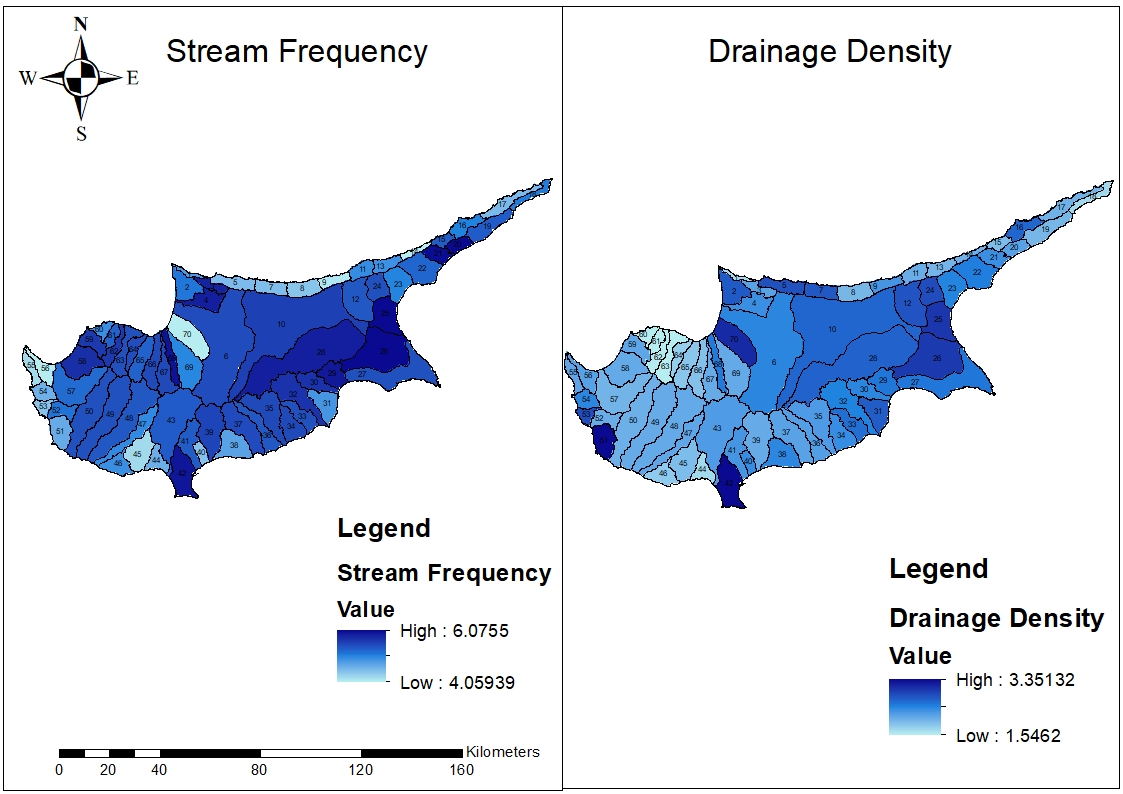


# **3. Morphometric risk by category (Shape, Scale, Topography and Hydrology)**

The values for each morphometric parameter were linearly standardized over the basins from 1 to 5 in accordance with their correlation to flood risk, denoting very low, low, medium, high, and very high flood potential, respectively. Additionally, the morphometric risks associated with each parameter type (i.e., scale, shape, topographic, and drainage network) were calculated by adding the normalized risks associated with each parameter falling into that category. This aggregation facilitated a secondary linear ranking of the cumulative values, enabling the ranking of the basins based on their morphometric risks. The flood potential scale risk map (S.M 2a) shows the basins with the highest risks, namely basins 6, 10, and 28. These basins were characterized by large basin areas of 737 km², 867 km², and 602 km², respectively, as well as perimeters of 165 km, 207 km, and 162 km, respectively. The combination of large basin areas and perimeters contributes to a higher flood potential in these particular basins. The flood potential shape risk map (S.M 2b) illustrates very high flood risk in the basins 12, 24, 23, 70, and 54 as they are characterized as the circular, less elongated, non-compacted nature basins, respectively. Basins 66 and 67 located to the west of Troodos mountains exhibited notably high reliefs, relief ratios, and ruggedness numbers compared to the other basins. These elevated topographic characteristics contribute to an increased flood risk within these basins, as evident in the flood potential topographic risk map (S.M 2c). The combination of high reliefs, relief ratios, and ruggedness numbers in these basins indicates a greater susceptibility to flooding. The flood potential drainage network map (S.M 2d) demonstrates significant high risks in Basins 6 and 10 due to the high number and order of streams. Additionally, these basins had the longest stream lengths and highest texture ratios, indicating the highest flood potential drainage risk among all the basins.

**S.M 3** Morphometric risk maps (a) scale risk, (b) shape risk, (c) topographic risk, (d) drainage network risk.

| **(a)**  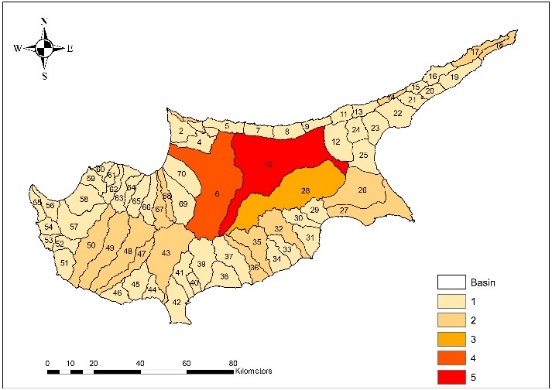 | **(b)**  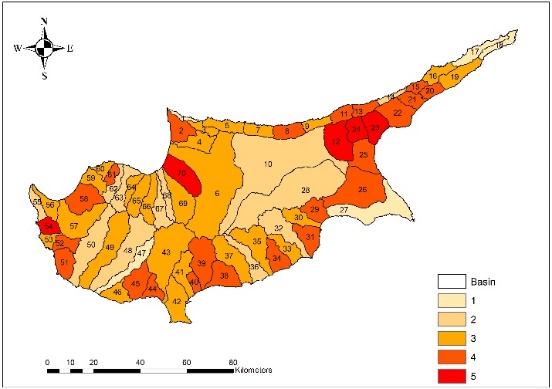 |
| --- | --- |
| **(c)**  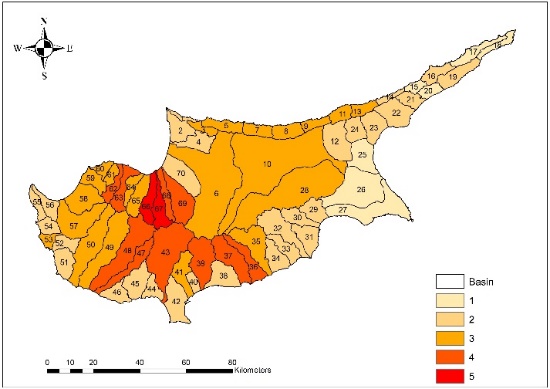 | **(d)**  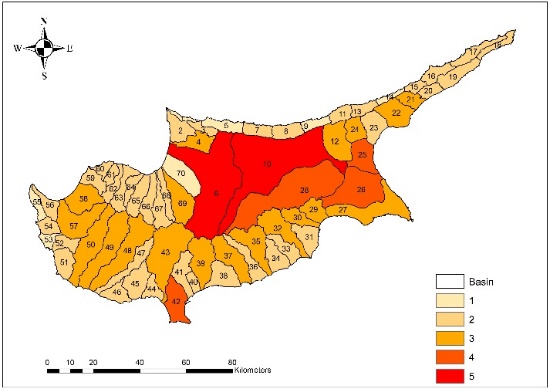 |

# **4. FR results**

**S.M 4** Frequency ratio results.

| Factor (Category) | | Features | Areal histogram (%) | Flood frequency (%) | FR |
| --- | --- | --- | --- | --- | --- |
| MR (G) | Very low risk  Low risk  Moderate risk  High risk  Very high risk | | 2.44  21.41  43.67  15.07  17.41 | 0  17.59  37.04  17.59  27.78 | 0  0.82  0.85  **1.17**  **1.6** |
| RRA (H) | 0 - 0.05  0.05 - 0.2  0.2 - 0.39  0.39 - 0.7  0.7 - 1.86  1.86 - 3.17  3.17 - 20.02 | | 26.82  10.93  32.55  9.25  14.81  1.62  4.01 | 13.89  3.7  43.52  7.41  24.07  1.85  5.56 | 0.52  0.34  **1.34**  0.8  **1.63**  **1.14**  **1.38** |
| CN (H) | 70 - 74  74 - 79 79 - 83  83 - 86  86 - 89  89 - 95  95 - 100 | | 32.14  7.99  24.23  4.54  18.61  11.2  1.29 | 3.7  4.63  73.15  2.78  9.26  6.48  0 | 0.12  0.58  **3.02**  0.61  0.5  0.58  0 |
| AS (T) | Flat  North  Northeast  East  Southeast  South  Southwest  West  Northwest  North | | 2.93  6.62  11.9  12.47  12.68  12.64  12.07  11.87  11.8  5.03 | 3.7  7.41  15.74  16.67  12.96  14.81  14.81  8.33  2.78  2.78 | **1.26**  **1.12**  **1.32**  **1.34**  **1.02**  **1.17**  **1.23**  0.7  0.24  0.55 |
| TWI (H) | 2.34 - 5.36  5.36 - 6.78  6.78 - 8.47  8.47 - 10.42  10.42 - 12.64  12.64 - 15.74  15.74 - 24.97 | | 25.35  31.74  23.13  10.19  5.78  2.96  0.86 | 10.19  42.59  26.85  13.89  3.7  1.85  0.93 | 0.4  **1.34**  **1.16**  **1.36**  0.64  0.63  1.08 |
| SPI (H) | -3.69 - -0.50  -0.50 - 1.1  1.1 - 2.54  2.54 - 3.84  3.84 - 5.51  5.51 - 8.02  8.02 - 15.69 | | 8.47  20.6  26.78  24  13.69  4.85  1.61 | 11.11  34.26  37.96  6.48  8.33  0.93  0.93 | **1.31**  **1.66**  **1.42**  0.27  0.61  0.19  0.58 |
| TC (H) | 0.05 - 0.39  0.39 - 0.68  0.68 - 1.05  1.05 - 1.55  1.55 - 2.28  2.28 - 3.46  3.46 - 6.77 | | 45.48  31.2  13.64  6.13  2.63  0.71  0.22 | 87.96  5.56  6.48  0  0  0  0 | **1.93**  0.18  0.48  0  0  0  0 |
| ProfC (T) | Upwardly convex  Linear  Upwardly concave | | 41.66  11.5  46.84 | 38.89  10.19  50.93 | 0.93  0.89  **1.09** |
| PlanC (T) | sideward concave  linear  sideward convex | | 38.18  19.87  41.94 | 32.41  25  42.59 | 0.85  **1.26**  **1.02** |
| DEM (T) | 0- 120  120- 266  266- 438  438- 641  641- 886  886- 1,223  1,223- 1,954 | | 34.78  25.64  15.25  11.37  7.51  4.29  1.15 | 38.89  28.7  11.11  12.96  3.7  3.7  0.93 | **1.12**  **1.12**  0.73  **1.14**  0.49  0.86  0.81 |
| Sl (T) | 0 - 1.22  1.22 -2.56  2.56 - 4.85  4.85 - 8.70  8.70 - 14.68  14.68 - 22.67  22.67 - 71.36 | | 14.59  15.04  14.1  14.08  14.07  14.08  14.05 | 54.63  27.78  7.41  3.7  5.56  0.93  0 | **3.75**  **1.85**  0.53  0.26  0.39  0.07  0 |
| AR (M) | 233 - 357  357 - 436  436 - 510  510 - 601  601 - 699  699 - 812  812 - 971 | | 4.72  24.53  26.91  15.54  17.13  7.00  4.17 | 9.26  34.26  24.07  19.44  5.56  3.70  3.70 | **1.96**  **1.40**  0.89  **1.25**  0.32  0.53  0.89 |
| DFR (H) | 0 - 48.18  48.18 - 103.78  103.78 - 159.37  159.37 - 211.25  211.25 - 266.84  266.84 - 337.25  337.25 - 945.05 | | 24.51  30.42  19.05  12.47  8.46  4.12  0.96 | 32.41  30.56  13.89  17.59  3.70  1.85  0.00 | **1.32**  **1.00**  0.73  **1.41**  0.44  0.45  0.00 |
| R20 (M) | 124 - 212  212 - 263  263 - 307  307 - 354  354 - 402  402 - 467  467 - 579 | | 4.88  28.16  19.81  15.03  10.54  15.16  6.42 | 11.11  35.19  20.37  14.81  10.19  5.56  2.78 | **2.28**  **1.25**  **1.03**  0.99  0.97  0.37  0.43 |

**Note: Bold indicates significant FR (>1)**

# **5. FR-SE results**

**S.M 5** FR-SE weights.

| Factor (Category) | | FR | $P_{ij}$ | $P_{\mathrm{ij}}\log_{2} P_{\mathrm{ij}}$ | $H_{j}$ | $W_{j}$ | Normalized  $W_{j}*Z/M_{i}$ |
| --- | --- | --- | --- | --- | --- | --- | --- |
|  | |  |  |  | $H_{jmax}$ |  |  |
|  | |  |  |  | $I_{j}$ |  |  |
|  | |  |  |  | $P_{j}$ |  |  |
| MR (G) | 0  0.82  0.85  **1.17**  **1.6** | | 0  0.19  0.19  0.26  0.36 | 0  -0.45  -0.46  -0.51  -0.53 | 1.94 | 0.14 | 0.13 |
|  |  | |  |  | 2.32 |  |  |
|  |  | |  |  | 0.16 |  |  |
|  |  | |  |  | 0.89 |  |  |
| RRA (H) | 0.52  0.34  **1.34**  0.8  **1.63**  **1.14**  **1.38** | | 0.07  0.05  0.19  0.11  0.23  0.16  0.19 | 15  -0.27  -0.21  -0.45  -0.35  -0.49  -0.42  -0.46 | 2.66 | 0.05 | 0.04 |
|  |  | |  |  | 2.81 |  |  |
|  |  | |  |  | 0.05 |  |  |
|  |  | |  |  | 1.02 |  |  |
| CN (H) | 0.12  0.58  **3.02**  0.61  0.5  0.58  0 | | 0.02  0.11  0.56  0.11  0.09  0.11  0 | -0.12  -0.35  -0.47  -0.36  -0.32  -0.35  0 | 1.95 | 0.24 | 0.15 |
|  |  | |  |  | 2.81 |  |  |
|  |  | |  |  | 0.31 |  |  |
|  |  | |  |  | 0.77 |  |  |
| AS (T) | **1.26**  **1.12**  **1.32**  **1.34**  **1.02**  **1.17**  **1.23**  0.7  0.24  0.55 | | 0.13  0.11  0.13  0.13  0.1  0.12  0.12  0.07  0.02  0.06 | -0.38  -0.35  -0.39  -0.39  -0.34  -0.36  -0.37  -0.27  -0.13  -0.23 | 3.21 | 0.03 | 0.02 |
|  |  | |  |  | 3.32 |  |  |
|  |  | |  |  | 0.03 |  |  |
|  |  | |  |  | 1 |  |  |
| TWI (H) | 0.4  **1.34**  **1.16**  **1.36**  0.64  0.63  1.08 | | 0.06  0.2  0.18  0.21  0.1  0.09  0.16 | -0.25  -0.47  -0.44  -0.47  -0.33  -0.32  -0.43 | 2.7 | 0.04 | 0.02 |
|  |  | |  |  | 2.81 |  |  |
|  |  | |  |  | 0.04 |  |  |
|  |  | |  |  | 0.94 |  |  |
| SPI (H) | **1.31**  **1.66**  **1.42**  0.27  0.61  0.19  0.58 | | 0.22  0.28  0.23  0.04  0.1  0.03  0.1 | -0.48  -0.51  -0.49  -0.2  -0.33  -0.16  -0.32 | 2.5 | 0.1 | 0.06 |
|  |  | |  |  | 2.81 |  |  |
|  |  | |  |  | 0.11 |  |  |
|  |  | |  |  | 0.86 |  |  |
| TC (H) | **1.93**  0.18  0.48  0  0  0  0 | | 0.75  0.07  0.18  0  0  0  0 | -0.31  -0.27  -0.45  0  0  0  0 | 1.03 | 0.23 | 0.15 |
|  |  | |  |  | 2.81 |  |  |
|  |  | |  |  | 0.63 |  |  |
|  |  | |  |  | 0.37 |  |  |
| ProfC (T) | 0.93  0.89  **1.09** | | 0.32  0.3  0.37 | -0.53  -0.52  -0.53 | 1.58 | 0.003 | 0.01 |
|  |  | |  |  | 1.58 |  |  |
|  |  | |  |  | 0.004 |  |  |
|  |  | |  |  | 0.97 |  |  |
| PlanC (T) | 0.85  **1.26**  **1.02** | | 0.27  0.4  0.33 | -0.51  -0.53  -0.53 | 1.57 | 0.01 | 0.02 |
|  |  | |  |  | 1.58 |  |  |
|  |  | |  |  | 0.01 |  |  |
|  |  | |  |  | 1.04 |  |  |
| DEM (T) | **1.12**  **1.12**  0.73  **1.14**  0.49  0.86  0.81 | | 0.21  0.19  0.13  0.15  0.06  0.11  0.14 | -0.47  -0.46  -0.39  -0.41  -0.25  -0.35  -0.39 | 2.73 | 0.02 | 0.02 |
|  |  | |  |  | 2.81 |  |  |
|  |  | |  |  | 0.03 |  |  |
|  |  | |  |  | 0.84 |  |  |
| Sl (T) | **3.75**  **1.85**  0.53  0.26  0.39  0.07  0 | | 0.55  0.27  0.08  0.04  0.06  0.01  0 | -0.48  -0.51  -0.28  -0.18  -0.24  -0.06  0 | 1.75 | 0.37 | 0.24 |
|  |  | |  |  | 2.81 |  |  |
|  |  | |  |  | 0.38 |  |  |
|  |  | |  |  | 0.98 |  |  |
| AR (M) | **1.96**  **1.40**  0.89  **1.25**  0.32  0.53  0.89 | | 0.27  0.19  0.12  0.17  0.04  0.07  0.12 | -0.51  -0.46  -0.37  -0.44  -0.2  -0.28  -0.37 | 2.63 | 0.07 | 0.04 |
|  |  | |  |  | 2.81 |  |  |
|  |  | |  |  | 0.06 |  |  |
|  |  | |  |  | 1.03 |  |  |
| DFR (H) | **1.32**  **1.00**  0.73  **1.41**  0.44  0.45  0.00 | | 0.25  0.19  0.14  0.26  0.08  0.08  0 | -0.5  -0.45  -0.39  -0.51  -0.3  -0.3  0 | 2.45 | 0.1 | 0.06 |
|  |  | |  |  | 2.81 |  |  |
|  |  | |  |  | 0.13 |  |  |
|  |  | |  |  | 0.76 |  |  |
| R20 (M) | **2.28**  **1.25**  **1.03**  0.99  0.97  0.37  0.43 | | 0.31  0.17  0.14  0.13  0.13  0.05  0.06 | -0.52  -0.44  -0.4  -0.39  -0.39  -0.22  -0.24 | 2.59 | 0.08 | 0.05 |
|  |  | |  |  | 2.81 |  |  |
|  |  | |  |  | 0.08 |  |  |
|  |  | |  |  | 1.04 |  |  |

Note: Bold indicates significant FR (>1)

# **6. F-AHP pairwise matrix**

**S.M 6** Flood susceptibility Fuzzy AHP pairwise comparison matrix.

| Factor | RRA | AR | MR | CN | SL | DEM | AS | Prof C | Plan C | TWI | SPI | DFR | TC | R20 |
| --- | --- | --- | --- | --- | --- | --- | --- | --- | --- | --- | --- | --- | --- | --- |
| RRA | 1 | 1 | 1/3 | 1/4 | 1/5 | 1/3 | 1 | 1/2 | 1/2 | 1/3 | 1/3 | 1/3 | 1/5 | 1 |
| AR | 1 | 1 | 1/2 | 1/4 | 1/6 | 1/3 | 1/2 | 1/2 | 1/2 | 1/3 | 1/3 | 1/5 | 1/7 | 1 |
| MR | 3 | 2 | 1 | 1/4 | 1/3 | 1/3 | 1/2 | 1/2 | 1/2 | 1/3 | 1/2 | 1/4 | 1/6 | 1 |
| CN | 4 | 4 | 4 | 1 | 1/3 | 3 | 6 | 3 | 3 | 3 | 3 | 3 | 1/3 | 6 |
| SL | 5 | 6 | 3 | 3 | 1 | 3 | 5 | 5 | 5 | 3 | 3 | 6 | 1 | 6 |
| DEM | 3 | 3 | 3 | 1/3 | 1/3 | 1 | 5 | 5 | 5 | 1/3 | 1/3 | 6 | 1/6 | 6 |
| AS | 1 | 2 | 2 | 1/6 | 1/5 | 1/5 | 1 | 1/3 | 1/3 | 1/6 | 1/6 | 1/5 | 1/9 | 3 |
| Prof C | 2 | 2 | 2 | 1/3 | 1/5 | 1/5 | 3 | 1 | 1 | 1/3 | 1/3 | 1/3 | 1/6 | 3 |
| Plan C | 2 | 2 | 2 | 1/3 | 1/5 | 1/5 | 3 | 1 | 1 | 1/3 | 1/3 | 1/3 | 1/9 | 3 |
| TWI | 3 | 3 | 3 | 1/3 | 1/3 | 3 | 6 | 3 | 3 | 1 | 1 | 1 | 1/6 | 3 |
| SPI | 3 | 3 | 2 | 1/3 | 1/3 | 3 | 6 | 3 | 3 | 1 | 1 | 1/3 | 1/6 | 3 |
| DFR | 3 | 5 | 4 | 1/3 | 1/6 | 1/6 | 5 | 3 | 3 | 1 | 3 | 1 | 1/6 | 3 |
| TC | 5 | 7 | 6 | 3 | 1 | 6 | 9 | 6 | 9 | 6 | 6 | 6 | 1 | 9 |
| R20 | 1 | 1 | 1 | 1/6 | 1/6 | 1/6 | 1/3 | 1/3 | 1/3 | 1/3 | 1/3 | 1/3 | 1/9 | 1 |

# **7. F-AHP weights**

**S.M 7** Classification of AHP factors and fuzzy weights.

| Factor (Category) | | Features | Reclassified features | Fuzzy Weights | Defuzzified Weight | Normalized  Weight |
| --- | --- | --- | --- | --- | --- | --- |
| MR (G) | Very low risk  Low risk  Moderate risk  High risk  Very high risk | | 1  2  3  4  5 | (0.01,0.03,0.06) | 0.03 | 0.03 |
| RRA (H) | 0 - 0.05  0.05 - 0.2  0.2 - 0.39  0.39 - 0.7  0.7 - 1.86  1.86 - 3.17  3.17 - 20.02 | | 7  6  5  4  3  2  1 | (0.01,0.02,0.05) | 0.03 | 0.02 |
| CN (H) | 70 - 74  74 - 79 79 - 83  83 - 86  86 - 89  89 - 95  95 - 100 | | 1  2  3  4  5  6  7 | (0.05,0.12,0.26) | 0.14 | 0.12 |
| AS (T) | Flat  North  Northeast  East  Southeast  South  Southwest  West  Northwest  North | | 8  5  9  10  4  6  7  3  1  2 | (0.01,0.02,0.04) | 0.02 | 0.02 |
| TWI (H) | 2.34 - 5.36  5.36 - 6.78  6.78 - 8.47  8.47 - 10.42  10.42 - 12.64  12.64 - 15.74  15.74 - 24.97 | | 1  2  3  4  5  6  7 | (0.03,0.07,0.16) | 0.09 | 0.08 |
| SPI (H) | -3.69 - -0.50  -0.50 - 1.1  1.1 - 2.54  2.54 - 3.84  3.84 - 5.51  5.51 - 8.02  8.02 - 15.69 | | 1  2  3  4  5  6  7 | (0.03,0.07,0.15) | 0.08 | 0.07 |
| TC (H) | 0.05 - 0.39  0.39 - 0.68  0.68 - 1.05  1.05 - 1.55  1.55 - 2.28  2.28 - 3.46  3.46 - 6.77 | | 7  6  5  4  3  2  1 | (0.15,0.24,0.43) | 0.27 | 0.23 |
| ProfC (T) | Upwardly convex  Linear  Upwardly concave | | 1  2  3 | (0.02,0.04,0.07) | 0.04 | 0.04 |
| PlanC (T) | sideward concave  linear  sideward convex | | 3  2  1 | (0.02,0.03,0.07) | 0.04 | 0.03 |
| DEM (T) | 0- 120  120- 266  266- 438  438- 641  641- 886  886- 1,223  1,223- 1,954 | | 7  6  5  4  3  2  1 | (0.04,0.08,0.16) | 0.09 | 0.08 |
| Sl (T) | 0 - 1.22  1.22 -2.56  2.56 - 4.85  4.85 - 8.70  8.70 - 14.68  14.68 - 22.67  22.67 - 71.36 | | 7  6  5  4  3  2  1 | (0.07,0.17,0.36) | 0.20 | 0.17 |
| AR (M) | 233 - 357  357 - 436  436 - 510  510 - 601  601 - 699  699 - 812  812 - 971 | | 1  2  3  4  5  6  7 | (0.01,0.02,0.04) | 0.03 | 0.02 |
| DFR (H) | 0 - 48.18  48.18 - 103.778  103.778 - 159.368  159.368 - 211.25  211.25 - 266.84  266.84 - 337.25  337.25 - 945.05 | | 7  6  5  4  3  2  1 | (0.03,0.07,0.14) | 0.08 | 0.07 |
| R20 (M) | 124 - 212  212 - 263  263 - 307  307 - 354  354 - 402  402 - 467  467 - 579 | | 1  2  3  4  5  6  7 | (0.01,0.02,0.03) | 0.02 | 0.02 |

# **8. Comparison between the different hazard weights**

F-AHP and FR-SE weights were directly obtained from the results, while the normalized sum of FR values for each parameter was utilized for the FR method. The results obtained from F-AHP, FR, and FR-SE assigned the weights ranging from 2% to 23%, 3% to 12%, and 1% to 24%, respectively. The different models exhibited a significant variation in the assigned weights. Particularly, F-AHP and FR-SE demonstrated more dispersed weights compared to FR.

By aggregating the weights for factors within each category, the significance of each category on the generated maps was determined. The general category, consisting only of MR, obtained the highest weight of 13% in FR-SE. Among the meteorological factors, FR demonstrated the most significant effect with a total weight of 18%, which was twice the weight assigned by FR-SE, the second most influential method with 9%. For hydrologic factors, F-AHP, FR, and FR-SE all assigned high weights of 59%, 41%, and 48%, respectively. Regarding topographic factors, FR exhibited the highest significance with a weight of 36%, closely followed by F-AHP and FR-SE with weights of 34% and 30%, respectively. These findings regarding the variation in weights align with similar studies on natural hazards documented in the literature (Agrawal and Dixit 2022; Wu, Chen, and Lu 2022).

**S.M 8** Weight comparison between used methods

# **9. Flood vulnerability F-AHP consistency matrix and weights**

**S.M 9** Flood vulnerability AHP pairwise comparison matrix.

| Factor | P | EV | UR | RVI | DFH |
| --- | --- | --- | --- | --- | --- |
| P | 1 | 5 | 9 | 4 | 3 |
| EV | 1/5 | 1 | 3 | 1/4 | 1/6 |
| UR | 1/9 | 1/3 | 1 | 1/4 | 1/6 |
| RVI | 1/4 | 4 | 4 | 1 | 1/2 |
| DFH | 1/3 | 6 | 6 | 2 | 1 |

**S.M 10** Flood vulnerability F-AHP weights.

| Factor (Category) | | Features | Reclassified features | Fuzzy Weights | Defuzzified Weight | Normalized Weight |
| --- | --- | --- | --- | --- | --- | --- |
| P (E) | 0 - 1  1 - 5  5 - 11  11 - 17  17 - 24  24 - 31  31 - 42 | | 1  2  3  4  5  6  7 | (0.26,0.48,0.82) | 0.52 | 0.47 |
| EV (E) | 0 - 238  238 - 680  680 - 813  813 - 979  979 - 1059  1059 - 1297  1297 - 1576 | | 1  2  3  4  5  6  7 | (0.04,0.07,0.11) | 0.07 | 0.07 |
| UR (E) | 0 - 0.19  0.19 - 0.54 0.54 - 0.96  0.96 - 1.83  1.83 - 3.74  3.74 - 8.52  8.52 - 16.81 | | 1  2  3  4  5  6  7 | (0.03,0.04,0.06) | 0.04 | 0.04 |
| RVI (C) | 1 - 3  3 - 5  5 - 6  6 - 7  7 - 9  9 - 12  12 - 14 | | 1  2  3  4  5  6  7 | (0.1,0.16,0.29) | 0.18 | 0.16 |
| DFH (C) | 0 - 6479  6479 – 12276  12276- 19097  19097 – 28645  28645- 42967  42967 - 61382 61382- 86958 | | 1  2  3  4  5  6  7 | (0.16,0.26,0.44) | 0.29 | 0.26 |
